# Rangewide occupancy of a flagship species, the Coastal California Gnatcatcher (*Polioptila californica californica*) in southern California: Habitat associations and recovery from wildfire

**DOI:** 10.1371/journal.pone.0306267

**Published:** 2024-07-05

**Authors:** Barbara E. Kus, Kristine L. Preston, Alexandra Houston

**Affiliations:** U.S. Geological Survey, Western Ecological Research Center, San Diego, California, United States of America; Western Carolina University, UNITED STATES

## Abstract

The Coastal California Gnatcatcher (*Polioptila californica californica*), a federally threatened species, is a flagship species for regional conservation planning in southern California (USA). An inhabitant of coastal sage scrub vegetation, the gnatcatcher has declined in response to habitat loss and fragmentation, exacerbated by catastrophic wildfires. We documented the status of gnatcatchers throughout their California range and examined post-fire recovery of gnatcatchers and their habitat. We used GIS to develop a habitat suitability model for Coastal California Gnatcatchers using climate and topography covariates and selected over 700 sampling points in a spatially balanced manner. Bird and vegetation data were collected at each point between March and May in 2015 and 2016. Presence/absence of gnatcatchers was determined during three visits to points, using area searches within 150 x 150 m plots. We used an occupancy framework to generate Percent Area Occupied (PAO) by gnatcatchers, and analyzed PAO as a function of time since fire. At the regional scale in 2016, 23% of the points surveyed were occupied by gnatcatchers, reflecting the effect of massive wildfires in the last 15 years. Similarly, PAO in the post-fire subset of points was 24%, with the highest occupancy in unburned (last fire <2002) habitat. Positive predictors of occupancy included percent cover of California sagebrush (*Artemisia californica*), California buckwheat (*Eriogonom fasciculatum*), and sunflowers (*Encelia* spp., *Bahiopsis laciniata*), while negative predictors included laurel sumac (*Malosma laurina*) and total herbaceous cover; in particular, non-native grasses. Our findings indicate that recovery from wildfire may take decades, and provide information to speed up recovery through habitat restoration.

## Introduction

Endangered species have long been threatened by habitat loss, degradation, and fragmentation associated with anthropogenic land use. Increasingly, wildfire is exacerbating these processes and poses a major threat to biodiversity worldwide [1]. In California (USA), a global biodiversity hotspot [2], wildfire has emerged in the last quarter century as a leading threat to coastal sage scrub habitat [3–6]. Among the inhabitants of sage scrub are several endemic plants and animals, including the Coastal California Gnatcatcher (*Polioptila californica*; “gnatcatcher”), a federally threatened species restricted to coastal sage scrub in southern California [7]. Although fire is a natural part of the sage scrub ecosystem, changes to the fire regime, such as increased frequency and intensity, challenge the persistence of coastal sage scrub habitat and associated species [8–10]. Catastrophic fires such as the Cedar fire in 2003, the Harris fire in 2007, and a complex of fires in May 2014 burned hundreds of thousands of hectares in San Diego County alone, destroying and degrading habitat required by California Gnatcatchers. The impacts of wildfire are exacerbated by postfire invasion of non-native grasses and other herbaceous vegetation [5, 11, 12] which reduces habitat suitability for gnatcatchers [13], and promotes future fires through its high flammability [4, 14].

The Coastal California Gnatcatcher (hereafter, California Gnatcatcher) has become established as a “flagship” species for conservation to protect coastal sage scrub habitat and its inhabitants, and is a covered species under several regional habitat conservation plans (HCPs), including the Multiple Species Conservation Program [15] and the Multiple Habitat Conservation Program in San Diego County [16], the Orange County Central and Coastal Subregion Conservation Plan [17], and the Western Riverside County Multiple Species Habitat Conservation Plan [18]. Effective management of gnatcatchers under these plans relies on periodic monitoring to determine population trends and evaluate the plans’ success in protecting the species. Historically, gnatcatcher monitoring has been limited in geographic scope, often at the level of individual preserves or local jurisdictions, and has employed differing methodologies; limiting the extent to which findings can be generalized across larger spatial scales and multiple populations. While satisfying the monitoring requirements of individual conservation plans, local monitoring cannot capture the full effects of landscape-scale phenomena like wildfire which often span jurisdictional boundaries that birds do not recognize, nor can it assess features like connectivity among conserved lands that facilitates dispersal, colonization, and gene flow. Gnatcatcher monitoring was expanded in the early 2000’s with a series of surveys by Winchell and Doherty [13, 19] who investigated occupancy in a study area spanning Orange and San Diego Counties. In further work, Winchell and Doherty [20] examined vegetation characteristics as predictors of gnatcatcher occupancy, colonization, and extinction in unburned habitat. These studies significantly advanced our understanding of gnatcatcher distribution and habitat requirements, yielding data to better protect and restore habitat to promote gnatcatcher viability. Our goal was to build on and expand these studies to encompass the entire range of the Coastal California Gnatcatcher, and to take advantage of the opportunity provided by several recent large wildfires to improve our understanding of the effects of fire on gnatcatchers and their habitat.

We designed and implemented a standardized protocol to address two related objectives. First, we sought to determine gnatcatcher occupancy at the regional scale, including habitat from throughout the species’ range in southern California, as well as in two subregions: Orange County and San Diego County, to address specific management objectives within those jurisdictions associated with the requirements of their HCPs. We did not examine other subregions because gnatcatcher abundance was insufficient for our analytical approach. Second, we collected vegetation data to better understand gnatcatcher-habitat associations that influence occupancy. In a parallel objective, we evaluated the effect of fire on gnatcatchers and their habitat in two ways. First, we compared occupancy and vegetation characteristics across regional and subregional sites varying in the length of time since the last fire. Second, we created a separate postfire dataset that categorized sites according to time since last fire and compared occupancy and vegetation characteristics across categories. Together, the results of these investigations create a baseline for future rangewide monitoring to track changes in habitat condition that affect gnatcatcher occupancy, and inform management to protect important ecological conditions and processes required for species persistence.

## Methods

### Study area

We surveyed for California Gnatcatchers in coastal sage scrub habitat within the U.S. portion of the species’ range in Ventura, Los Angeles, San Bernardino, Riverside, Orange and San Diego counties, California. To establish a sampling frame for southern California, we developed a habitat suitability model [21] to use in place of a model (Technology Associates International Corporation [22] used in previous California Gnatcatcher surveys of coastal regions [13, 19], but not designed for inland locations which differ considerably from the coast in climatic and topographic conditions. We used Geographic Information System (GIS) software to create a grid of points oriented north to south that encompassed the entire southern California study area including developed lands and open space, with each point falling within the center of a 150-m (meter) x 150-m grid cell. We used ArcGIS and digital data layers to calculate various climatic, topographic, land use and vegetation variables at each point in the landscape grid. California Gnatcatchers are often associated with California sagebrush (*Artemisia californica*; e.g. [19]); however, our vegetation layers did not identify coastal sage scrub supporting California sagebrush for the entire study area. Thus, we modelled California sagebrush habitat suitability [23] and included sagebrush model output among the environmental variables calculated for each grid point.

We used a partitioned Mahalanobis D^2^ approach [23–27] to construct alternative models of habitat suitability for gnatcatchers in southern California. Mahalanobis D^2^ represents a standardized distance between the multivariate mean for environmental variables at locations where a species occurs and values calculated for the same set of environmental variables at each grid point in the landscape being modelled [25, 26]. The more similar environmental characteristics are at a point in the landscape to the species’ multivariate mean, the more suitable the habitat is for the species. Habitat suitability for each 150-m x 150-m grid cell in the study area is indicated by a Habitat Similarity Index (HSI) value that ranges from 0 (least similar to occupied habitat and considered least suitable) to 1 (most similar to occupied habitat and most suitable). We categorized habitat suitability for gnatcatchers based on HSI values as: Very High: 0.75–1.00, High: 0.50–0.74, Moderate: 0.25–0.49, and Low: 0–0.24.

We compiled California Gnatcatcher location records for 2000 to 2013 from a variety of sources including the U.S. Geological Survey, California Department of Fish and Wildlife’s California Natural Diversity Database, U.S. Fish and Wildlife Service (USFWS) Carlsbad Office, County of San Diego (SanBIOS), Center for Natural Lands Management, Marine Corps Air Station Miramar, Marine Corps Base Camp Pendleton, Naval Weapons Station Fallbrook, Natural Communities Coalition (formerly the Nature Reserve of Orange County), and Western Riverside County Multiple Species Habitat Conservation Program, and used these to develop and evaluate the performance of alternative habitat models. Records with spatial accuracy lower than 80 m radius were excluded to avoid characterizing habitat in cells where the birds did not occur. To characterize the environment used by gnatcatchers, we used ArcGIS to spatially join each gnatcatcher location to the center point of the closest cell in the landscape grid. We screened for spatial redundancy (location records that were assigned the same grid cell) and used only one gnatcatcher record per cell. We used 1,063 location records from multiple datasets to construct the models and 3,205 records from the USFWS database to independently evaluate and compare the performance of alternative habitat suitability models. To avoid spatially biased sampling, we employed a subsampling strategy to balance gnatcatcher locations used in constructing the models [28]. We divided the region up into 5 sampling units: Los Angeles/Ventura, Riverside/San Bernardino, Orange, San Diego Coastal, and San Diego Inland. We randomly subsampled 50 gnatcatcher locations from each area (i.e., a total of 250 gnatcatcher locations) and constructed a model. We repeated this subsampling for 1,000 iterations and then averaged the results to develop a final model from which the Mahalanobis D^2^ values were calculated across the landscape.

**Fig 1:**
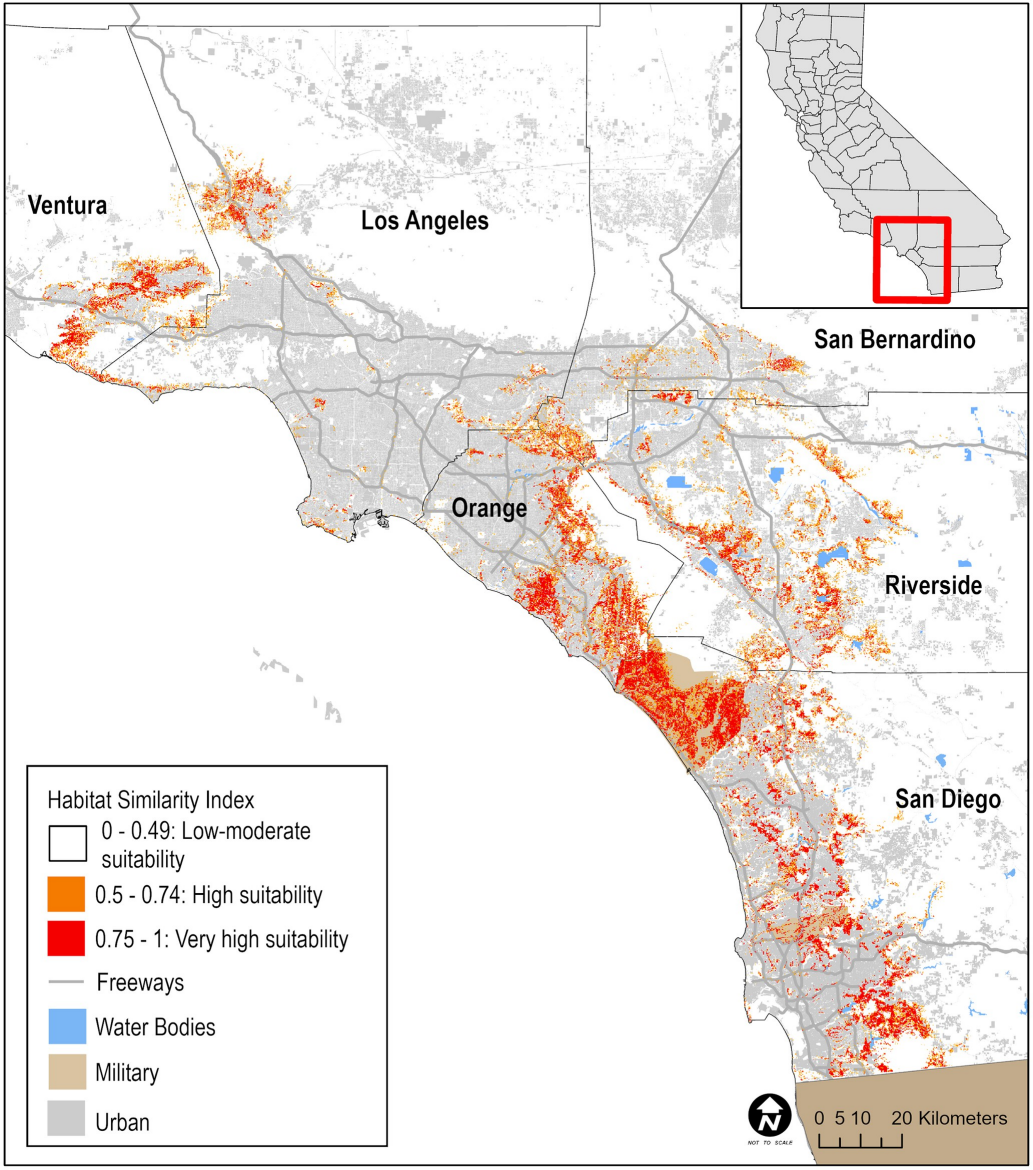
Map of California Gnatcatcher habitat in Ventura, Los Angeles, San Bernardino, Orange, Riverside, and San Diego counties as predicted by a habitat suitability model. Sources: [21, 29, 30].

Eighteen models with different combinations of variables were developed and their performance in predicting suitable habitat for California Gnatcatchers evaluated. The top-performing model included average minimum January and maximum July temperatures, annual precipitation, elevation, northness, eastness, slope, topographic heterogeneity, the percent of urban, coastal sage scrub and chaparral land cover within the 150-m x 150-m grid cell, and predicted habitat suitability for California sagebrush. We used this model to generate HSI values for each cell in the landscape grid, and used cells with HSI values ≥ 0.5 (Very High or High suitability; hereafter, “suitable”) to define our sampling frame (Fig 1).

**Fig 2:**
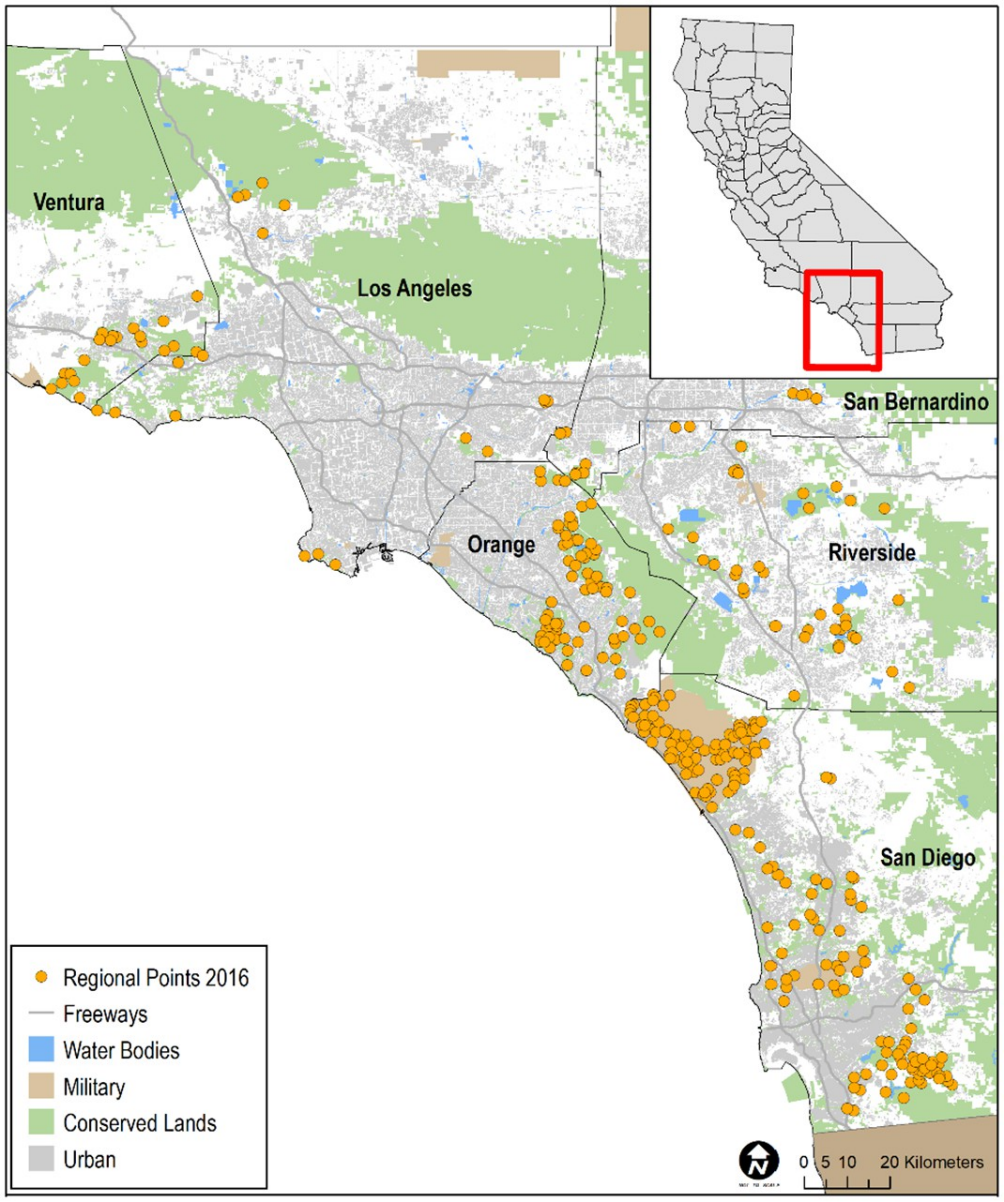
Distribution of California Gnatcatcher survey points at the regional scale in Ventura, Los Angeles, San Bernardino, Orange, Riverside, and San Diego counties, CA in 2016. Sources: [29–32].

**Fig 3:**
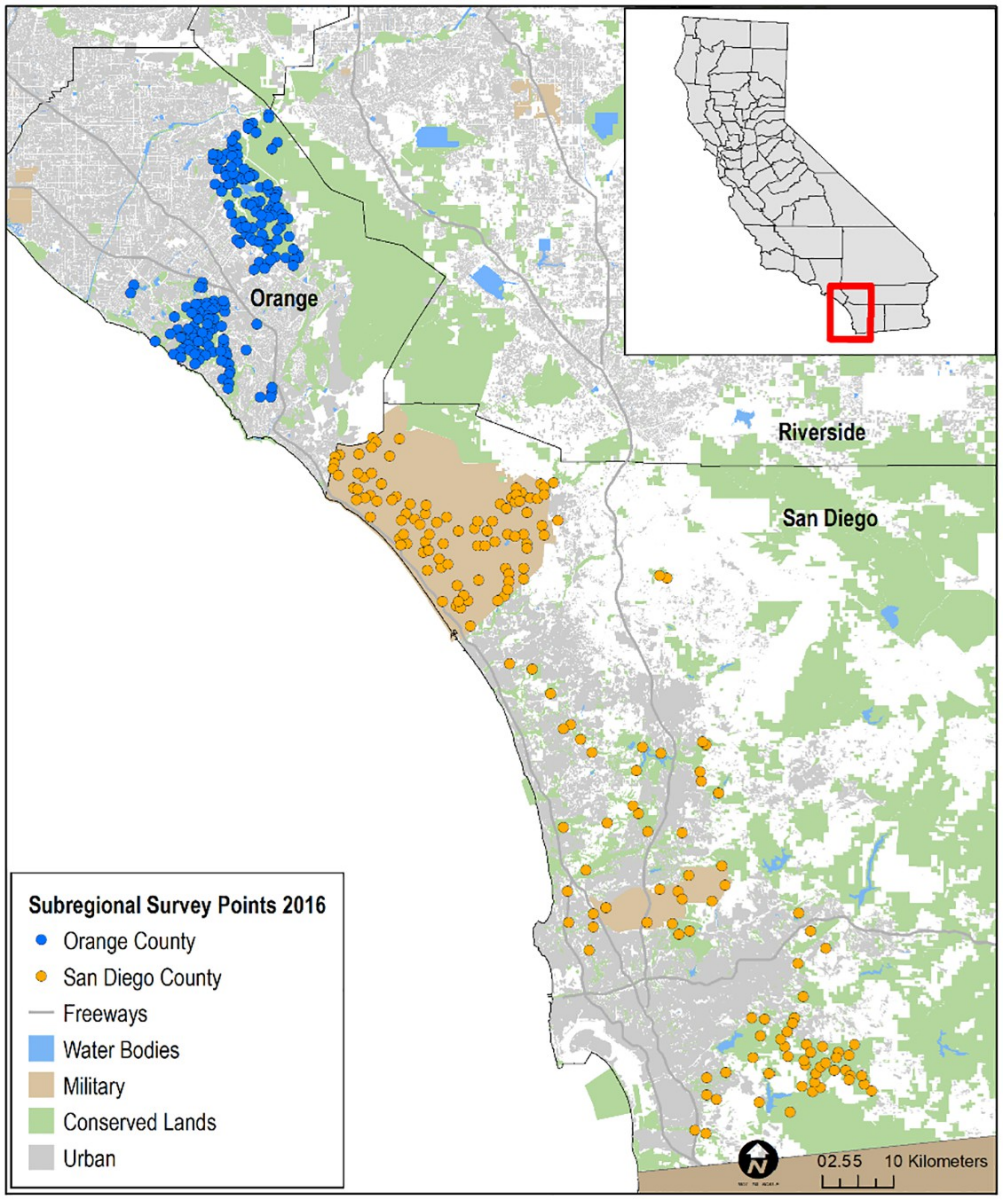
Distribution of California Gnatcatcher survey points at the subregional scale in San Diego and Orange counties, CA in 2016. Sources: [29–32].

**Fig 4:**
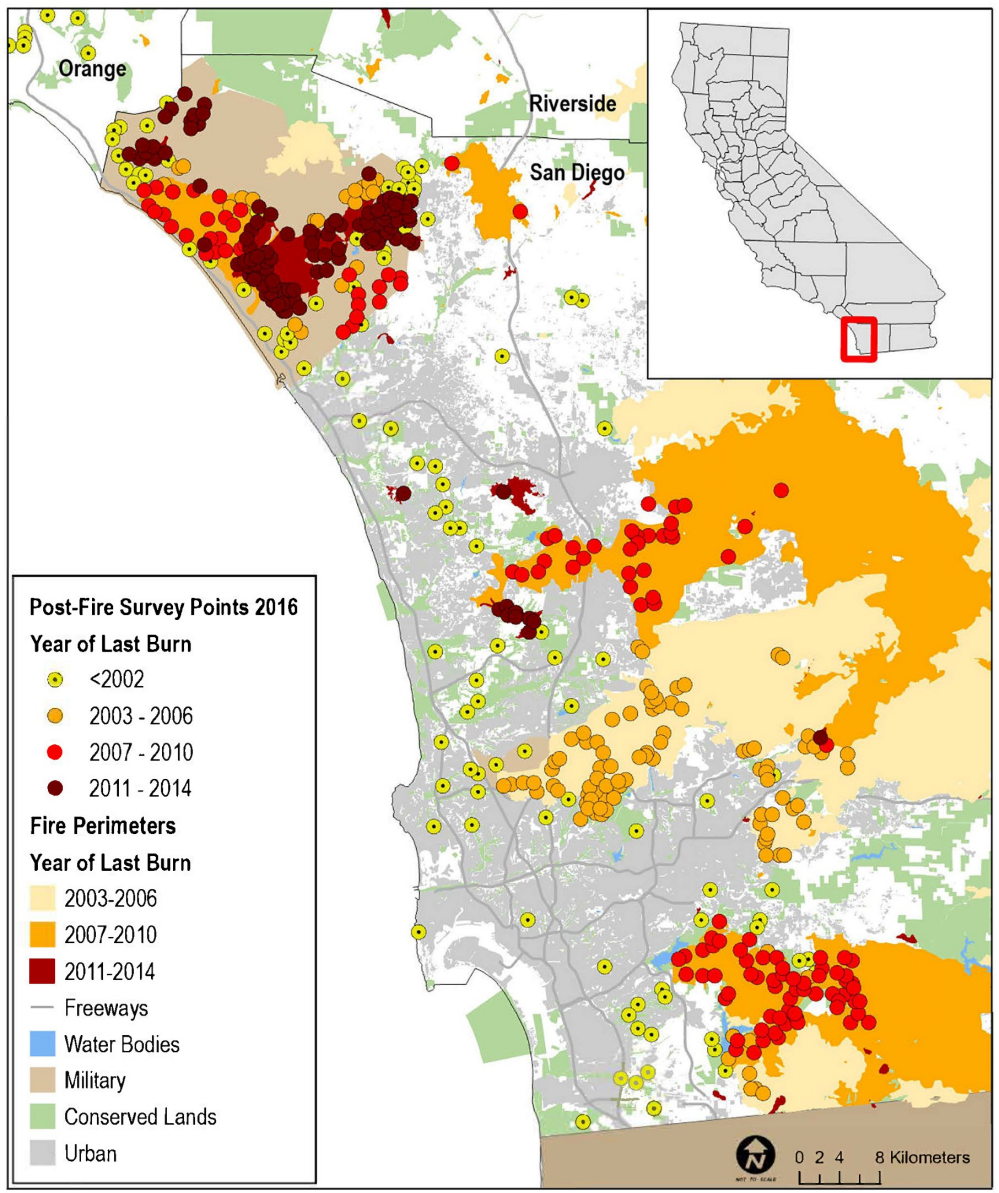
Distribution of California Gnatcatcher survey points by year of last fire in San Diego County, CA in 2016. Sources: [29–33].

We selected points for the regional survey from suitable habitat on conserved and military lands throughout southern California (Fig 2), and for the subregional surveys from suitable habitat in Orange and San Diego counties (Fig 3). Postfire points were restricted to San Diego County, and were selected from within the footprints of wildfires that burned in 2003–06, 2007–10, and 2011–14, periods that bracketed the three major recent fires of 2003, 2007, and 2014, respectively. A fourth category in the postfire study, that of “unburned”, was comprised of points selected from suitable habitat in San Diego County that had last burned between 2002 and 1878, the earliest year for which digital fire records are available [33; Fig 4]. The median year of the last fire in the unburned category was 1878.

### Sampling point selection

Sampling points for the regional, subregional, and postfire analyses were selected in the same manner unless otherwise noted.

#### Number of points

We ran simulations in Program MARK [34] to estimate the number of points needed to detect specific changes in California Gnatcatcher occupancy between successive surveys. For these simulations, we set gnatcatcher detection probability at 0.6, a conservative estimate based on a prior gnatcatcher survey with similar methodology [35]. Occupancy was set at 0.30, the mean occupancy in “high” and “very high” quality habitat surveyed in 2004, 2007, and 2009 (range = 0.24–0.39; [13]). We specified detection of a 30 percent change in gnatcatcher occupancy as the goal for the regional monitoring, and a 40 percent change for the subregional monitoring. Lower power to detect change at the subregional level was considered an acceptable way to manage cost and practicalities of subregional surveys given that they were conducted within the context of regional monitoring designed to detect regionally significant changes in occupancy that would trigger management response [18, 36]. Based on the simulations, we established sample sizes of 330 points for the regional survey and 180 for each subregional survey.

In a similar manner, we determined sample sizes for each of the four categories in the postfire study. Guided by previous surveys for gnatcatchers at burned sites [13], we estimated occupancy at 0.35 for unburned habitat, 0.30 for habitat burned in 2003–06, 0.15 for habitat burned in 2007–10, and 0.05 for habitat burned in 2011–14. We then evaluated simulations for sample sizes needed to provide power to distinguish among the four postfire categories with 90 and 95 percent confidence. Limited by the total number of points possible given the size of the burned areas, we opted for sample sizes of 110 points in each category. These sample sizes provided a power of 1.0 to detect a difference between occupancies of 0.05 (burned in 2011–14) and 0.35 (unburned) with 90 percent confidence. Power of other comparisons between burned and unburned points was 0.67 for occupancies of 0.15 (burned in 2007–10) and 0.35, and 0.09 for occupancies of 0.30 (burned in 2003–06) and 0.35. Among burned categories, power was 0.38 and 0.48 to detect differences between occupancies of 0.05 and 0.15, and 0.15 and 0.30, respectively.

#### Distribution of points

Survey points for the regional, subregional, and postfire components were selected separately in ArcGIS using the Spatially Balanced Sampling tool [37]. Spatially balanced sampling creates a more flexible and efficient design than random sampling for population trend analysis, and is robust to unanticipated events requiring adjustment to the design, such as loss of access to points, need to replace points, etc. [37, 38]. Points in the regional and subregional surveys were spaced at least 600 m apart to avoid double-counting birds [19]. Points in the postfire study were separated by a minimum of 450 m in order to achieve our desired samples sizes within the fire footprints.

#### Number and timing of visits

With an estimated occupancy probability of 0.3 and detectability of 0.6, 2 visits per point are sufficient to estimate gnatcatcher occupancy (Table 6.1 in MacKenzie et al. [39]). We conservatively chose to make 3 visits to each point during the first year of surveys in case detectability and occupancy differed substantially from our estimates. Points were surveyed at 2-week intervals between March 15 and April 30, 2016, coinciding with the pre-breeding and early breeding season when gnatcatcher vocal detectability is at a seasonal high [40]. Points burned in 2003–06, 2007–10, and 2011–14 were also surveyed during the same timeframe in 2015 in a pilot study of postfire plots.

### Data collection

#### Bird surveys

Gnatcatcher detection-nondetection surveys were conducted within 150-m x 150-m (2.25 ha) plots centered on each sampling point [35]. Surveys were performed between dawn and late morning/early afternoon depending on weather, avoiding conditions of excessive wind (> 20 km/h), temperatures below 4.5 degrees C or above 32 degrees C, or precipitation greater than a drizzle. Upon arriving at the plot, the surveyor recorded date and start time, and accessed imagery on hand-held devices displaying their location and the plot boundaries in ArcGIS Collector. After allowing 1–2 minutes for activity at the plot to settle, the surveyor walked slowly and methodically through the entire plot, looking and listening for gnatcatchers. Playbacks of gnatcatcher “mew” vocalizations were broadcast in a standardized manner [35] from each of the 4 cardinal quadrants of the plot (northwest, northeast, southeast, southwest). A single bout of songs lasting approximately 20 seconds was played in each quadrant, with no more than 4 bouts per plot. Broadcasts were directed towards the center of the plot to avoid attracting birds from outside the plot. Surveys ended after 45 minutes, or when an adult California Gnatcatcher was detected within the plot, at which time the surveyor recorded the location of any birds detected, the number of song broadcasts (0–4), and the survey end time.

#### Vegetation sampling

Vegetation data were collected at each survey plot between May 1 and August 2, 2016. We used a modified point intercept method to record data at 2-m intervals along 2 perpendicular (north-south and east-west) 30-m transects centered within each of the 4 cardinal quadrants of the plot, for a total of 128 vegetation sampling points per plot. Field surveyors used high resolution digital aerial imagery and a compass to navigate to transects and estimate sampling locations along their length. At each sampling point, surveyors placed a 2-m long measuring pole perpendicular to the ground and recorded “hits” of shrubs, trees, and herbs. Shrubs and trees were defined by their height at the location of the measuring pole, with shrubs being < 2 m and trees ≥ 2 m tall. In addition to hits of shrubs and trees, surveyors recorded the associated height of the tallest shrub and tree at the point. Based on preliminary analysis of vegetation data collected previously in suitable gnatcatcher habitat [20], we recorded species for hits of 20 shrub, tree and herbaceous species thought to influence gnatcatcher occupancy and habitat suitability (Table 1); all other species were combined into “other shrub/tree” or “other herbaceous” categories and the species for woody vegetation noted under comments. We recorded “Dead” for hits of woody vegetation where the entire plant was dead. At points where no vegetation occurred, substrate was recorded as bare ground, boulder (large rock too heavy to lift), or pavement.

**Table 1:** Vegetation species, heights, and unvegetated substrates recorded at California Gnatcatcher survey plots in 2016.

Species/Substrate	Taxonomic Name/Comments	Code
**Shrub/Tree:**		
Oak	*Quercus* spp.	QUER
Laurel sumac	*Malosma laurina*	MALA
Elderberry	*Sambucus mexicana*	SAMX
Lemonadeberry	*Rhus integrifolia*	RHIN
Lilac	*Ceanothus* spp.	CEAN
California sagebrush	*Artemisia californica*	ARCA
California buckwheat	*Eriogonum fasciculatum*	ERFA
Bush sunflower	*Encelia californica*	ENCA
Brittlebush	*Encelia farinosa*	ENFA
San Diego sunflower	*Bahiopsis laciniata*	BALA
White sage	*Salvia apiana*	SAAP
Black sage	*Salvia mellifera*	SAME
Coyote bush	*Baccharis pilularis*	BAPI
Deerweed	*Acmispon glaber*	ACGL
Yucca	*Hesperoyucca whipplei* or *Yucca* sp.	YUCC
Dead	Entire plant dead	DEAD
Other shrub/tree		OTHSHRTRE
Shrub height	Height of tallest shrub	SHRBHT
Tree height	Height of tallest tree	TREEHT
**Herbaceous:**		
Mustard	*Brassica nigra*, *B*. *tournefortii*	BRAS
Star thistle	*Centaurea melitensis*	CEME
Artichoke thistle	*Cynara cardunculus*	CYCA
Fennel	*Foeniculum vulgare*	FOVU
Non-native grasses	*Bromus* spp., *Avena* spp., others	GRASS
Other herbaceous		OTHHRB
**Substrate:**		
Bare ground		BARE
Boulder		BOULDER
Pavement		PAVEMENT

Trees: height ≥ 2m; shrubs: height < 2m. Substrate only recorded when no vegetation present at sampling point.

### Data analysis

We calculated percent cover for each vegetation variable (Table 1) as the percent of the 128 sampling points at which the species or substrate occurred, and then averaged these over the four cardinal quadrants to obtain an overall average for each gnatcatcher survey plot. Similarly, we calculated average shrub and tree heights for each gnatcatcher survey plot. In 12 plots for which shrub height was missing, we used the average shrub height calculated for the rest of the plots in that dataset as the value for shrub height. In addition to the species and substrates recorded in the field, we analyzed five species extracted from “Comments” for woody vegetation recorded as “other shrub” or “other tree” after input from field investigators and data exploration revealed these species to be more common in the northern part of the gnatcatcher’s range than in San Diego County from which the vegetation list (Table 1) was derived. These species included chamise (*Adenostoma fasciculatum*, “ADFA”), chaparral bushmallow *(Malacothamnus fasciculatus*, “MAFA”), Menzie’s goldenbush (*Isocoma menziesii*, “ISME”), purple sage (*Salvia leucophylla*, “SALE”), and cactus (Cactaceae, primarily *Opuntia* sp. and *Cylindropuntia* sp., “CACTUS”).

We created two composite variables combining structurally similar species. “Sunflowers” (SUNFL) included bush sunflower, brittlebush, San Diego sunflower, and Menzie’s goldenbush, while “Sage” (SAGE) combined black and purple sages. These composite variables allowed us to analyze taxonomically or structurally similar species that differed in distribution across the study area. We created a variable called “Total shrub/tree” (TOTSHRTRE) that represented cover of all species (i.e., the 25 individual species plus “other” species). In calculating total shrub/tree cover, each point received a maximum of 1 “hit”, regardless of how many species of shrubs or trees occurred there. We present means and standard deviations (SD) to display variability among plots, and present means and standard errors (SE) in comparisons across datasets where non-overlapping SEs indicate significant differences.

In addition to analyzing vegetation variables, we used ArcGIS to extract physical variables for each gnatcatcher survey plot in the regional and subregional datasets, including distance to the Pacific coast (DISTCOAST), elevation (ELEV), and slope (SLOPE) of the plot location. We did not analyze physical variables for the postfire dataset because points were distributed non-randomly with regard to these variables; recent fires were concentrated at lower elevations near the coast while older fires were inland at higher elevations. We also calculated an index of time since the last fire at each regional and subregional plot (LASTFIRE) relative to 1878, the earliest record in the fire perimeter dataset [33]. LASTFIRE was calculated by subtracting 1877 from the year of the most recent fire at each plot to generate values that ranged from 1 (last fire in or prior to 1878) to 137 (last fire in 2014). Structuring the index as increasing with recency of fire rather than decreasing eliminated the need to recalculate LASTFIRE in future survey datasets while retaining the ability to update the variable for any plots that burned subsequent to the 2016 survey.

We modelled single season occupancy in Program MARK [34] to estimate detection and occupancy probabilities (*p* and psi, respectively) separately for 2015 and 2016. The analysis for 2015 included estimates of *p* and psi for the postfire dataset, while those for 2016 included estimates for the regional, 2 subregional, and postfire datasets. We modelled occupancy as a function of covariates (see below), and evaluated support for models using Akaike’s Information Criterion corrected for small sample size (AIC_c_; [41]). We tested for goodness-of-fit using the parametric bootstrapping approach of MacKenzie and Bailey [42] in Package Unmarked [43], and present adjusted estimates of the overdispersion parameter (ĉ) and QAIC_c_ values for overdispersed datasets. We modelled detection probability as constant among surveys.

We identified covariates for inclusion in models of occupancy for each dataset using a 3-step screening process. First, we performed a Principal Components Analysis (PCA) using function Principal with varimax rotation to identify patterns of variability in vegetation composition and structure across the survey plots. The PCA for postfire models was performed on the unburned plots to provide a reference condition for burned plots. To evaluate whether habitat variability among plots was related to occupancy by gnatcatchers, we used the scores of individual plots on each principal component as input for two inferential analyses. We ran t-tests to compare means of plots with and without gnatcatcher detections (hereafter, “occurrence” or “presence”), and used GLM (generalized linear model; binomial family) to evaluate the principal components as significant predictors of gnatcatcher occurrence. Principal components significantly (P ≤ 0.05) related to gnatcatcher occurrence in either t-tests or GLMs were evaluated further. We assessed the contribution of variables to each significant principal component and extracted those with loadings ≥ 0.35 to create a set of covariates for inclusion in models of occupancy.

Models were built hierarchically by first creating models based on each significant component from the PCA and comparing them using AIC. The top-ranked model at this step was refined and evaluated for support by adding covariates from the best-supported models for the other principal components. Finally, the physical covariates were added to the top vegetation model to produce a final model set with ΔAIC_c_ or ΔQAIC_c_ ≤ 4. We removed models with uninformative parameters (i.e., where addition or removal of a covariate had a negligible effect on deviance; [44]).

We present the final model set for each of the datasets (regional, subregional, and postfire), and considered models within 2 AIC_c_ or QAIC_c_ units of the top-ranked model to be the best supported [41]. We estimated occupancy for each dataset by averaging over the best-supported models. We used the “group” function in Program MARK [34] to estimate occupancy for each of four postfire categories: unburned, 2003–2006 burned, 2007–2010 burned, and 2011–2014 burned. Relationships between covariates and estimated occupancy are plotted based on the top model for each dataset, or the best-supported model in which that particular covariate appears.

Data analyzed in this paper are available in a U.S. Geological Survey data release [45]. This research was observational in nature. The field study was approved by the U.S. Geological Survey Western Ecological Research Center Animal Care and Use Committee.

## Results

We surveyed for gnatcatchers at 334 regional points (Table 2). Six points were dropped following the first survey, 3 points were excluded from analysis because none of the surveys of these points met the 45-minute protocol in the absence of gnatcatcher detections, and 2 points lacked vegetation data. One hundred and eighty Orange County subregional points were surveyed; a landslide at 1 point prevented collection of vegetation data, so 179 points were analyzed. The San Diego subregional dataset included 178 points after excluding 2 points dropped after the first visit and 2 points failing to meet the survey time requirement. Among the postfire datasets, the number of points surveyed for gnatcatchers ranged from 103 to 111. Of 103 unburned points, 1 was dropped after the first survey. Of 111 points burned in 2007–10, 2 points failed to meet the survey time requirement and were excluded from analysis. Eighty-seven to ninety-nine percent of points in each dataset were surveyed 3 times (Table 2).

**Table 2:** Sample sizes for points surveyed and analyzed in 2015 and 2016 regional, subregional and postfire datasets.

Dataset	# points surveyed	# points excluded	# points analyzed	# points per survey frequency		
				3	2	1
Regional	334	11	323	285	32	6
OC Subregional	180	1	179	172	7	0
SD Subregional	182	4	178	174	2	2
Postfire:						
Unburned	103	1	102	101	0	1
2003–06 burned	107	1	106	105	1	0
2007–10 burned	111	2	109	106	2	1
2011–14 burned	106	0	106	103	3	0

### Vegetation composition

#### Regional and subregional points

**Fig 5:**
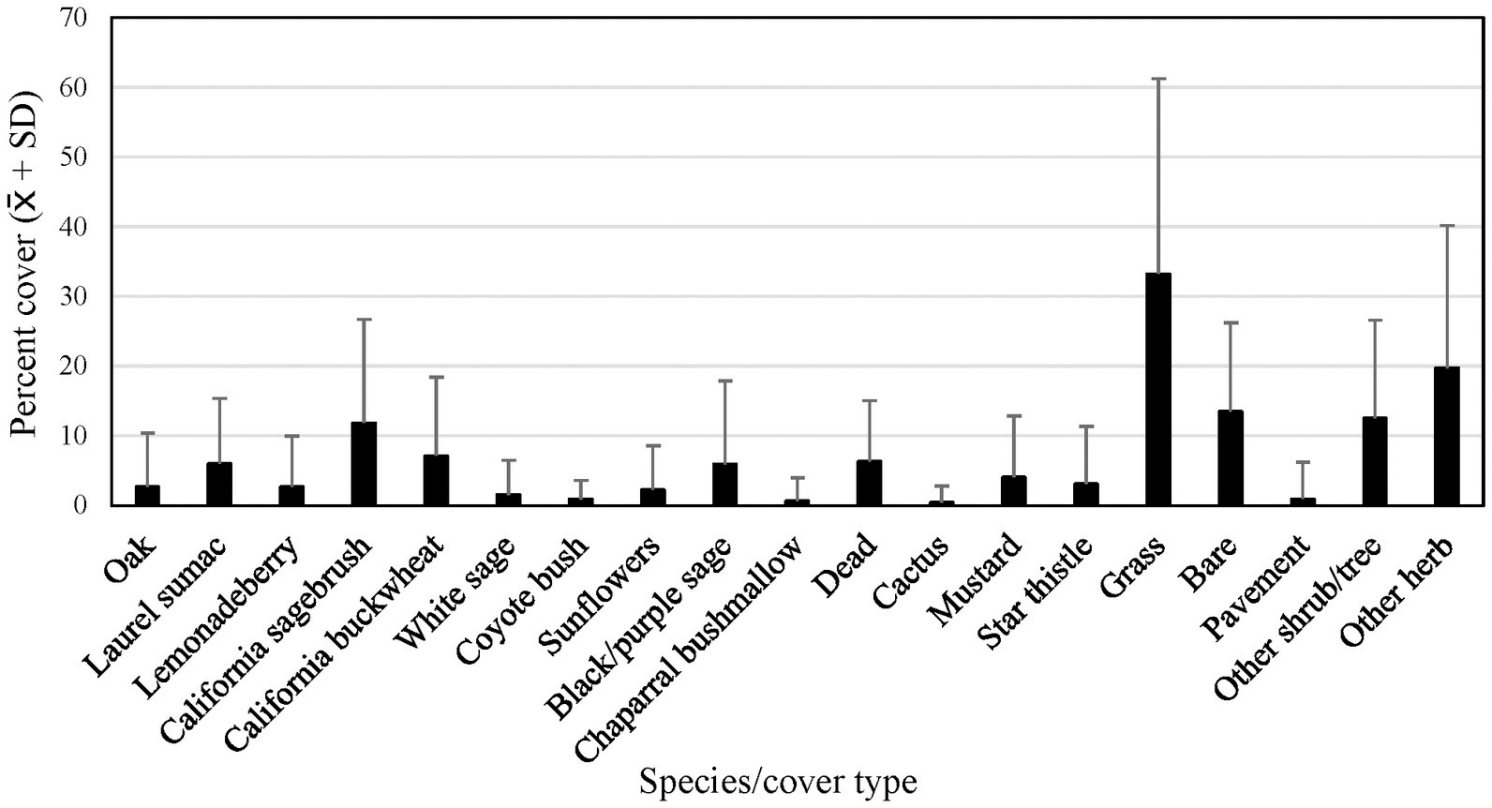
Average percent cover (+ SD) of vegetation species and cover types at regional points, 2016. See Table 2 for sample sizes.

We analyzed 17 species and cover types that averaged at least 1 percent of the cover at either regional or subregional points (Fig 5). At regional points, grass and other herbaceous vegetation made up most of the cover. Among woody shrubs and trees, California sagebrush and California buckwheat contributed the most cover, followed by black/purple sage and laurel sumac. Bare ground averaged 14 percent (± 13 percent SD) of cover at regional points.

**Fig 6:**
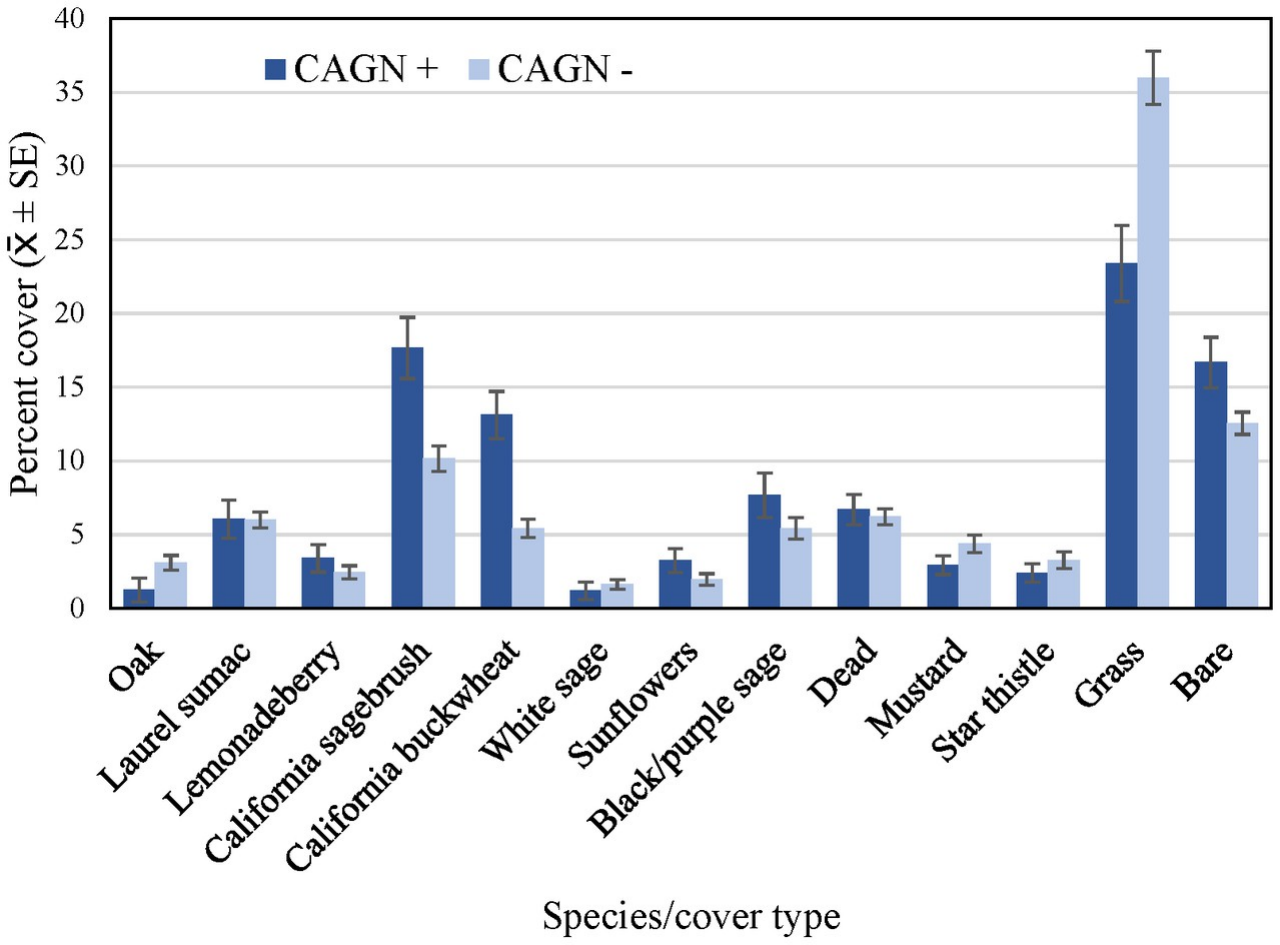
Average percent cover (± SE) of vegetation and cover types at regional points occupied and not occupied by California Gnatcatchers, 2016. See Table 2 for sample sizes.

Gnatcatcher occurrence at points was non-random with regard to vegetation composition (Fig 6). Cover of California sagebrush, California buckwheat, black/purple sage, sunflowers, and bare ground was higher on average at points with gnatcatcher detections than at points without detections. Points without gnatcatcher detections supported higher cover of grass, mustard, and oaks than points where gnatcatchers were detected. These differences were observed in both the Orange County and San Diego subregions, with the exception that oak cover did not differ between points with and without gnatcatcher detections in San Diego.

**Fig 7:**
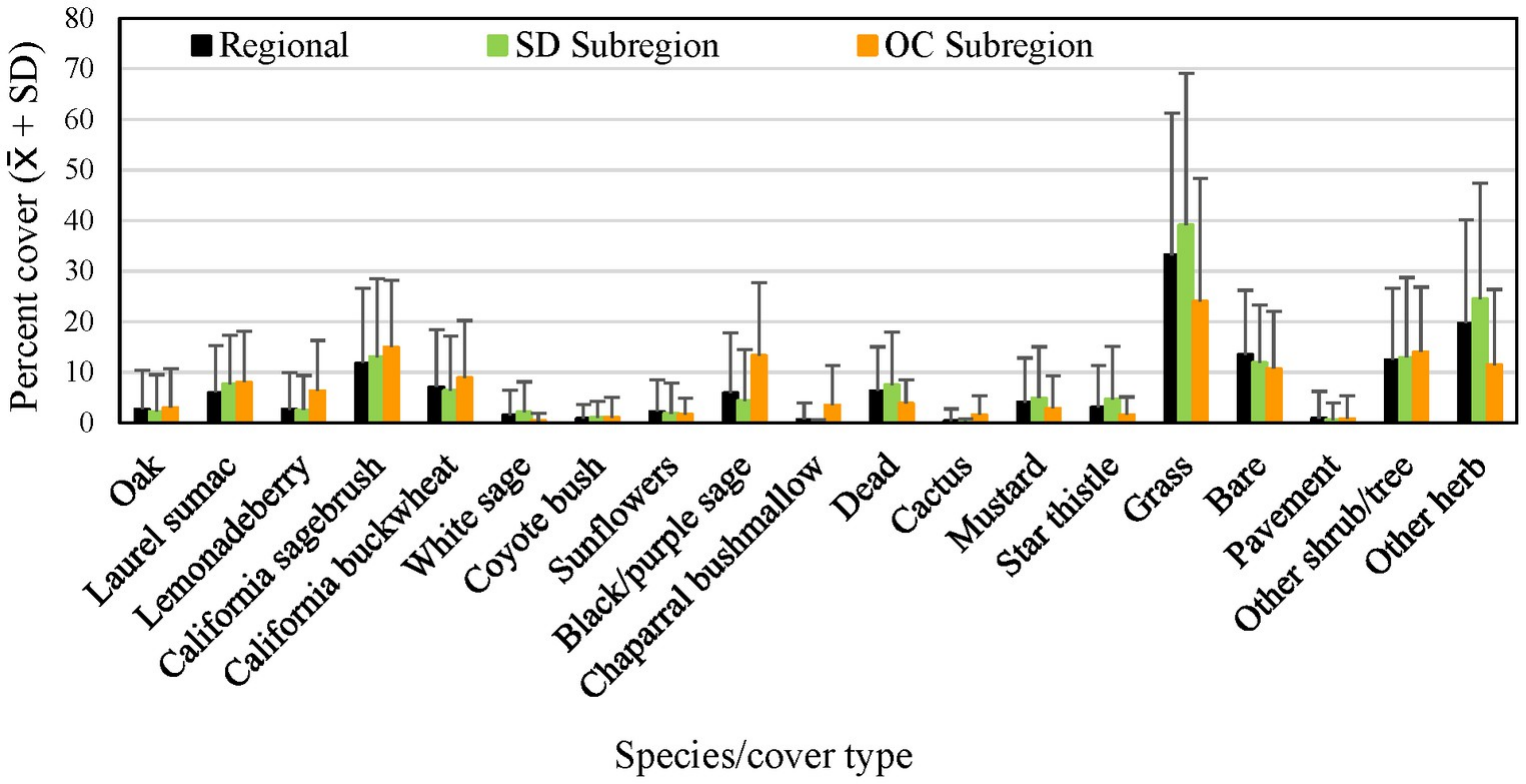
Average percent cover (+ SD) of vegetation species and cover types at regional, San Diego (SD) subregional, and Orange County (OC) subregional points, 2016. See Table 2 for sample sizes.

**Fig 8:**
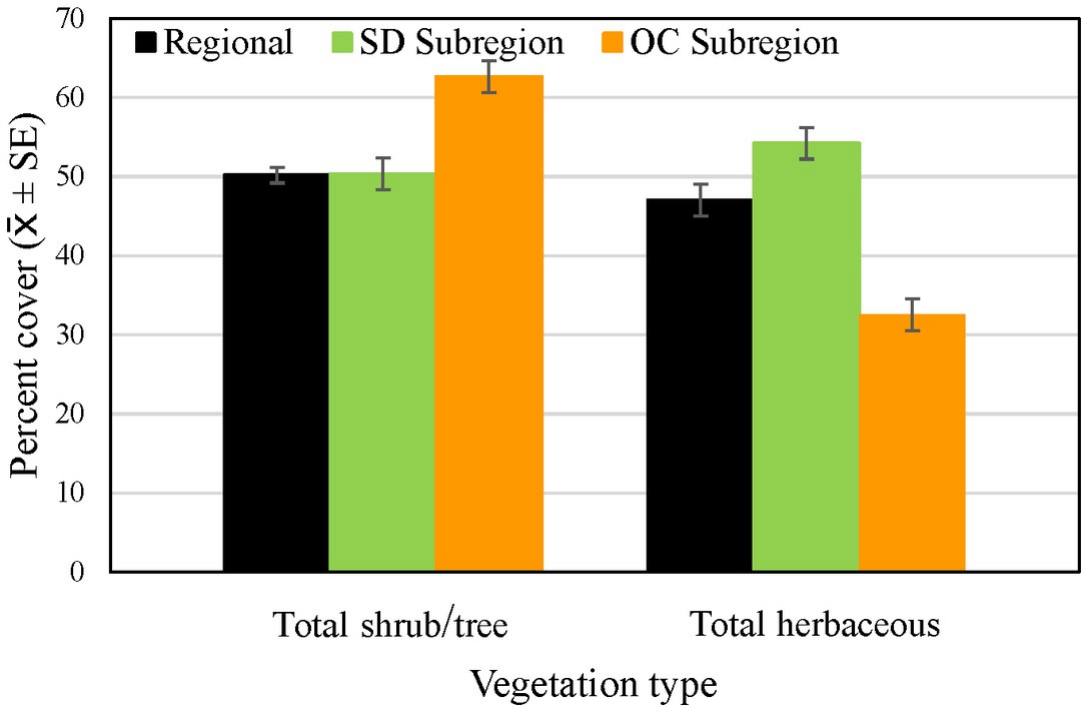
Average percent cover (± SE) of total shrubs and trees, and total herbaceous vegetation, across regional, San Diego (SD) subregional, and Orange County (OC) subregional points, 2016. See Table 2 for sample sizes.

Of the species making up the most cover across regional and subregional points, average cover of grass and other herbaceous vegetation, as well as dead woody vegetation, was lower in the Orange County subregion than in the San Diego sub-region and the region as a whole (Fig 7), while cover of California sagebrush, California buckwheat, and black/purple sage tended to be higher. Total shrub and tree cover, including species making up < 1 percent of cover, was higher in Orange County, and total herbaceous cover lower, than at the regional and San Diego subregional points (Fig 8).

#### Postfire points

**Fig 9:**
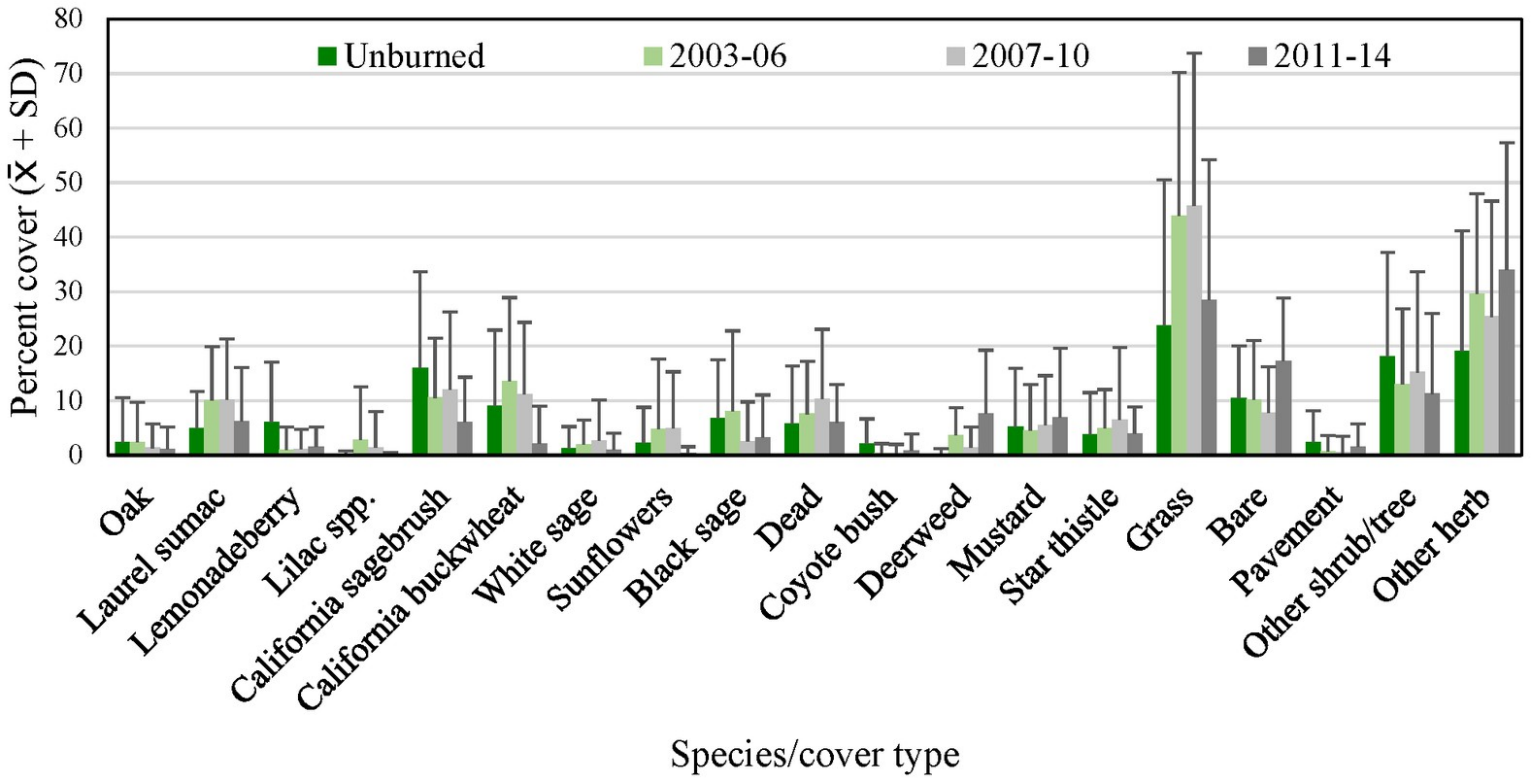
Average percent cover (+ SD) in 2016 of vegetation species and cover types at unburned points and points burned in 2003–06, 2007–10, and 2011–14. See Table 2 for sample sizes.

Vegetation at the unburned points was similar to that in the region as a whole (Fig 9). Grass and other herbaceous species made up most of the vegetation, although average cover of grass at unburned points (24 percent) was roughly two-thirds that at regional points (33 percent, Fig 5). California sagebrush and California buckwheat dominated the shrub cover at unburned points (Fig 9), as at regional and subregional points (Figs 7 and 8).

Fire altered the vegetation, which differed across points as a function of time since burn (Fig 9). At the most recently burned points (2011–14), cover of bare ground, grass and other herbaceous species, including fire-followers deerweed and black mustard, was higher than at unburned points. Among shrubs, cover of California sagebrush, California buckwheat, and black/purple sage was lower than at unburned points; in contrast, cover of laurel sumac was slightly higher.

At sites burned in 2007–10, grass cover was even higher than at the recently burned points, and approximately twice as high as in unburned habitat, while cover of bare ground was reduced relative to recently burned sites (Fig 9). Cover of other herbaceous species was lower than at the recently burned points, but still substantially higher than in unburned habitat. California sagebrush cover was twice as high as at recently burned points, but still lower than at unburned points. California buckwheat cover was over 5 times higher than at recently burned points, and exceeded the average in unburned habitat. Cover of laurel sumac was 60 percent higher, and double that in unburned habitat.

Differences in vegetation structure relative to unburned habitat persisted even into the oldest fire category (2003–06). Grass and other herbaceous cover remained elevated at approximately twice that in unburned habitat, as was the case for laurel sumac (Fig 9). California buckwheat cover continued to increase and exceeded that in unburned habitat. Cover of California sagebrush changed little relative to that at points last burned in 2007–10, and remained at about 60 percent of that at unburned points. In contrast, cover of black/purple sage was roughly 3 times higher than in the two younger fire categories, comparable to that at unburned sites.

**Fig 10:**
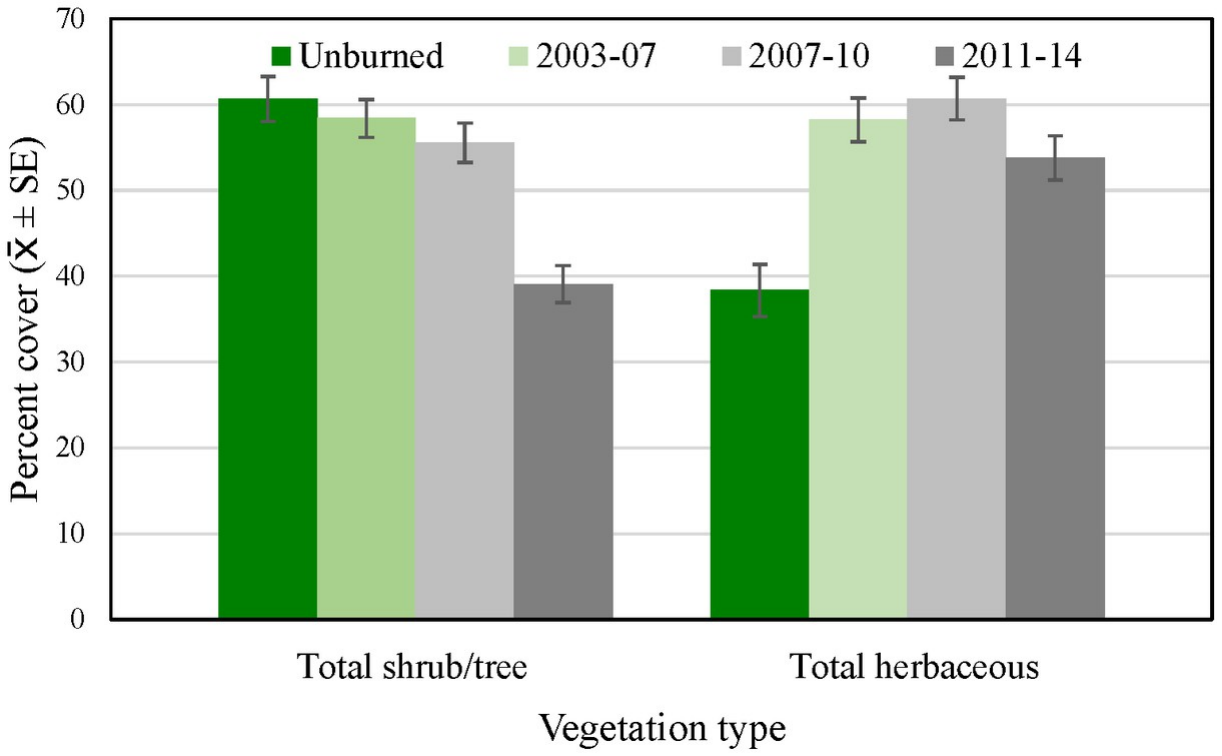
Average percent cover (± SE) in 2016 of total shrubs and trees, and total herbaceous vegetation, across unburned points and points burned in 2003–06, 2007–10, and 2011–14.

Overall, recently burned sites supported much more total herbaceous cover (including grass) than unburned sites, a difference that intensified and persisted over all three burn categories (Fig 10). In contrast, total shrub and tree cover was reduced to about two-thirds of that in unburned habitat immediately following fire, but recovered relatively quickly, although the recovery included species such as laurel sumac not necessarily associated with gnatcatcher occurrence.

### California Gnatcatcher occupancy

#### Habitat covariates

A Principal Components Analysis of vegetation data collected at regional points produced five Principal Components (PC) collectively explaining 37 percent of the variability across points (Table 3). PC1, which accounted for 10 percent of the variability, differentiated between sites with high cover of total shrub/tree and sage (especially black sage), as well as relatively tall shrubs, and low cover of grass, other herbaceous, and total herbaceous vegetation. PC2, which accounted for 7 percent of the variability, described habitat characterized by high cover of oaks, Mexican elderberry, other shrub/tree, total shrub/tree, and tall tree and shrub heights, with little bare ground and low cover of brittlebush. PC3 (7 percent of variability) described habitat suggestive of disturbed coastal sage scrub, including high cover of California sagebrush, white sage, lilac, laurel sumac, grass and star thistle, and low cover of brittlebush and bare ground. PC4 (7 percent of variability) was suggestive of less disturbed coastal sage scrub supporting high cover of California sagebrush, California buckwheat, sunflowers (especially San Diego sunflower), laurel sumac, dead, and total shrub/tree. PC5 (6 percent of variability) described a more limited type of sage scrub characterized by high cover of chamise, deerweed, yucca, and bare ground, and low cover of grass and other herbaceous vegetation.

**Table 3:** Variables with loadings > 0.35 on factors extracted by Principal Components Analysis of the regional, San Diego and Orange County subregional, and postfire (unburned) datasets for 2016.

**Regional**	**PC1**	**PC2**	**PC3**	**PC4**	**PC5**
	+ SAME	+ SAMX	+ ARCA	+ ARCA	+ ADFA
	+ SAGE	+ QUER	+ CEAN	+ ERFA	+ ACGL
	+ TOTSHRTRE	+ OTHSHRTRE	+ CEME	+ MALA	+ YUCC
	- GRASS	+ TOTSHRTRE	+ MALA	+ BALA	+ BARE
	- OTHHRB	- ENFA	+ SAAP	+ SUNFL	- GRASS
	- TOTHRB	- BARE	+ GRASS	+ DEAD	- TOTHRB
	+ SHRBHT	+ SHRBHT	- ENFA	+ TOTSHRTRE	
		+ TREEHT	- BARE		
Var. explained	0.10	0.07	0.07	0.07	0.06
Effect in GLM	+++			++	
P value t-test	<0.001			0.03	
**San Diego Subregional**	**PC1**	**PC2**	**PC3**	**PC4**	**PC5**
	+ ARCA	+ FOVU	+RHIN	+ SAME	+ ARCA
	+ ERFA	+ GRASS	+SAMX	+ SAGE	+ CEAN
	+ MALA	+ OTHHRB	+QUER	- TOTHRB	+ CEME
	+ BALA	+ TOTHRB	+ OTHSHRTRE		+ MALA
	+ SUNFL	- BARE	+ TOTSHRTRE		+ SAAP
	+ DEAD	- TOTSHRTRE	+ SHRBHT		+ TOTSHRTRE
	+ TOTSHRTRE		+ TREEHT		
			+ PAVEMENT		
			- BARE		
Var. explained	0.09	0.08	0.08	0.08	0.07
Effect in GLM	+	-		+	+
P value t-test	0.14	0.01		0.12	0.08
**Orange County Subregional**	**PC1**	**PC2**	**PC3**	**PC4**	**PC5**
	+ ARCA	+ BAPI	+ SAMX	+ ADFA	+ ACGL
	+ SAME	+ ENCA	+ QUER	+ MAFA	+ CEME
	+ SAGE	+ ISME	+ OTHSHRTRE	+ SAME	+ SAAP
	+ ERFA	+ SUNFL	- SAME	+ SAGE	+ OTHHRB
	+ MALA	+ SAMX	- SAGE	+ YUCC	+ TOTHRB
	+ RHIN		+ TREEHT	+ OTHSHRTRE	- BARE
	+ TOTSHRTRE			+ BOULDER	
	- BRAS				
	- CYCA				
	- GRASS				
	- OTHHRB				
	- TOTHRB				
	+ SHRBHT				
Var. explained	0.15	0.07	0.07	0.06	0.06
Effect in GLM	++	++		-	-.
P value t-test	0.01	0.001		0.01	
**Postfire**	**PC1**	**PC2**	**PC3**	**PC4**	**PC5**
	+ RHIN	+ ARCA	+ ERFA	+ SAME	+ FOVU
	+ SAMX	+ CEME	+ ENCA	+ SAGE	- ACGL
	+ OTHSHRTRE	+ MALA	+ BALA	- ARCA	- ISME
	+ TOTSHRTRE	+ SAAP	+ SUNFL	- CEME	- BARE
	- GRASS	+ DEAD		- SAAP	+ SHRBHT
	- OTHHRB	+ TOTSHRTRE		- TOTHRB	
	- TOTHRB	- BARE			
	+ SHRBHT	+ SHRBHT			
Var. explained	0.10	0.08	0.07	0.07	0.05
Effect in GLM	++		++		
P value t-test	0.01		<<0.001	0.05	

A “+” preceding variable name indicates a positive loading, while “-”indicates a negative loading. Effect in GLM (generalized linear model): +++ = positive effect on gnatcatcher presence, P≤ 0.001; ++ = positive effect, P≤ 0.01; + = positive effect, P≤ 0.05;— = negative effect, P≤ 0.05; -. = negative effect, P≤ 0.10. Vegetation codes (in alphabetical order): ACGL = *Acmispon glaber* (deerweed), ADFA = *Adenostoma fasciculatum* (chamise), ARCA = *Artemisia californica* (California sagebrush), BALA = *Bahiopsis laciniata* (San Diego sunflower), BARE = bare ground, SAGE = black/purple sage, BOULDER = boulder, BRAS = *Brassica* spp. (mustard), CEAN = *Ceanothus* spp. (lilac), CEME = *Centaurea melitensis* (star thistle), DEAD = dead, ENCA = *Encelia californica* (California sunflower), ENFA = *E*. *farinosa* (brittlebush), ERFA = *Eriogonum fasciculatum* (California buckwheat), FOVU = *Foeniculum vulgare* (fennel), ISME = *Isocoma menziiesii* (Menzie’s goldenbush), GRASS = non-native grass, MAFA = *Malacothamnus fasciculatus* (chaparral bushmallow), MALA = *Malosma laurina* (laurel sumac), OTHHRB = other herbaceous, OTHSHRTRE = other shrub/tree, PAVEMENT = pavement, QUER = *Quercus* spp. (oak), RHIN = *Rhus integrifolia* (lemonadeberry), SAAP = *Salvia apiana* (white sage), SAME = *S*. *mellifera* (black sage), SAMX = *Sambucus mexicana* (Mexican elderberry), SHRBHT = shrub height, SUNFL = California sunflower, San Diego sunflower, brittlebush, Menzie’s goldenbush), TOTHRB = total herbaceous, TOTSHRTRE = total shrub/tree, TREEHT = tree height, YUCC = *Hesperoyucca whipplei* or *Yucca* sp.

Analysis of the San Diego subregion, Orange County subregion, and postfire datasets produced PCs similar to those for the regional dataset, although not always ordered the same in terms of the amount of variability explained (Table 3). GLMs regressing gnatcatcher presence against scores on each factor for points within the four datasets consistently identified factors with low grass and other herbaceous cover, and high sage (SAME and SAGE) cover (PC1, PC4, and PC1 in the regional, San Diego subregional, and Orange County subregional datasets, respectively) as significant positive predictors of gnatcatcher occurrence. In the postfire dataset, the conditions of low grass and herbaceous cover, and high sage (SAME and SAGE) cover, were split across two PCs (PC1 and PC4, respectively); only PC1 was found to be a significant predictor of gnatcatcher presence, but average scores on PC4 differed between points with and without gnatcatcher detections in t-tests. Cover of California sagebrush, California buckwheat, and sunflowers were also positively associated with gnatcatcher occurrence at points (PC4 in the regional dataset, PC1 in the San Diego subregional dataset, PC1 and PC2 in the Orange County subregional dataset, PC3 in the postfire dataset). Negative predictors were found in analyses of the San Diego and Orange County subregional datasets, and included factors describing sites with little bare ground and high cover of herbaceous vegetation, in particular exotic species such as fennel (San Diego subregional, PC2) and star thistle (Orange County subregional, PC5). An additional factor in the Orange County subregional dataset, PC4, emerged as a negative predictor of gnatcatcher presence; this factor is suggestive of rocky chapparal habitat with high cover of chamise, sage (SAME and SAGE), chaparral bushmallow, and other shrubs/trees.

### California Gnatcatcher occupancy models

#### Regional

**Fig 11:**
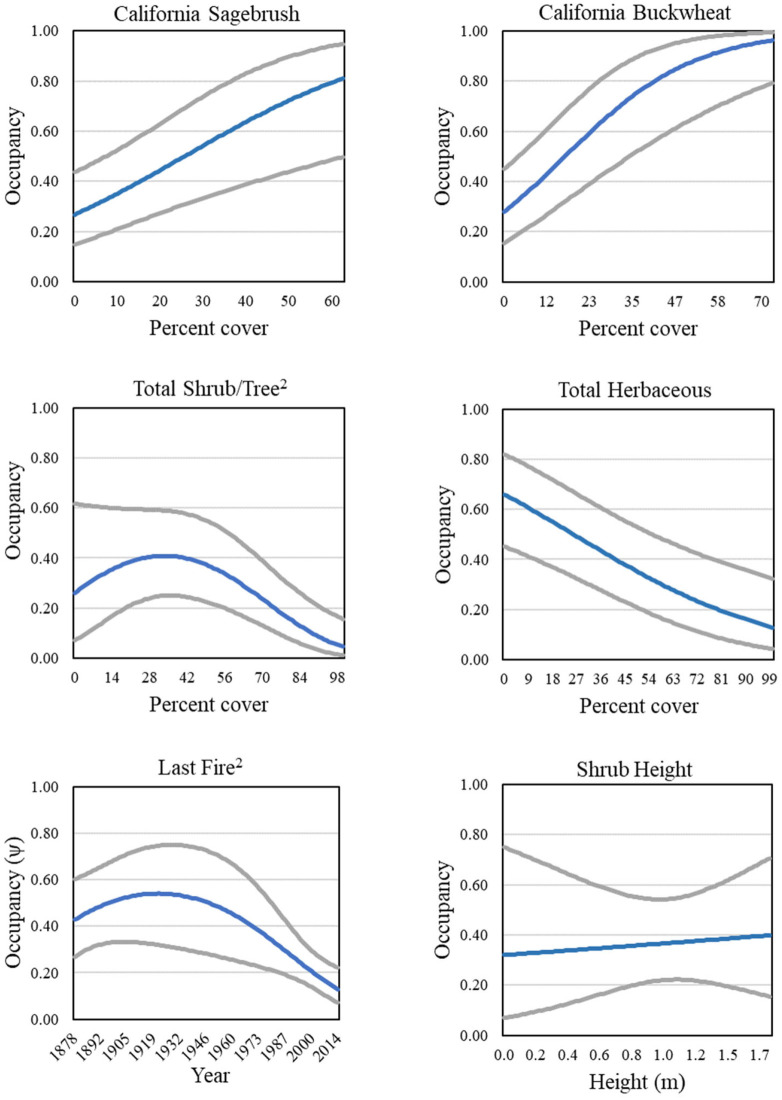
California Gnatcatcher occupancy in relation to cover of California sagebrush, California buckwheat, total shrub/tree (quadratic function), total herbaceous, time since last fire (quadratic function; plotted as year of last fire), and shrub height at regional points in 2016. Gray lines represent 95% confidence interval. All covariates except shrub height from model 1; shrub height from model 2; Table 4).

Two models predicting gnatcatcher occupancy at the regional scale were well-supported (ΔAIC_c_ ≤ 2, Tables 4 and 8). Cover of California sagebrush and California buckwheat appeared in both models, and both covariates were positive predictors of occupancy (Fig 11). Total shrub and tree cover also appeared in both models, with the results suggesting a threshold response whereby occupancy increased with increasing cover of shrubs and trees up to about 30–40 percent, then declined thereafter (Fig 11). There was some support for shrub height as an additional although weak positive predictor of gnatcatcher occupancy (Fig 11). In contrast, total herbaceous cover, which appeared in all models, was negatively related to occupancy (Fig 11). Occupancy increased with time since the last fire over a period of about 80 years before plateauing, based on the top model (Fig 11).

**Table 4:** Logistic regression models of California Gnatcatcher occupancy in 2016 as a function of habitat covariates at the regional scale.

	Model	ΔAIC_c_	AIC_c_ Weight	logLik	Number Parameters	Deviance
1	**ERFA**+**ARCA**+**TOTSHRTRE**^**2**^+**TOTHRB**+**LASTFIRE**^**2**^	0.00	0.66	1.00	9	531.91
2	**ERFA**+**ARCA**+SHRBHT+**TOTSHRTRE**^**2**^+**TOTHRB**+**LASTFIRE**^**2**^	2.07	0.24	0.36	10	531.85
3	**ERFA**+**ARCA**+SHRBHT+TOTSHRBTRE^**2**^+**TOTHRB**+**LASTFIRE**	3.77	0.10	0.15	9	535.67

Detection probability (*p*) constant across models. Models are ranked from best to worst based on Akaike’s Information Criterion for small samples (AIC_c_), the difference between the model’s AIC_c_ and the highest-ranked model’s AIC_c_ (ΔAIC_c_), and AIC_c_ weights. AIC_c_ is based on -2 x log_e_ likelihood (logLik) and the number of parameters in the model. Minimum AIC_c_ = 552.55. Only models with ΔAIC_c_ ≤ 4 presented. Bold means 95% confidence interval of the beta estimate for covariate does not include 0. ERFA = California buckwheat, ARCA = California sagebrush, TOTSHRTRE = total shrub/tree cover, TOTHRB = total herbaceous cover, LASTFIRE = time since last fire, SHRBHT = shrub height. A superscript 2 in a covariate code represents the quadratic (squared) function of that covariate.

#### San Diego subregion

**Fig 12:**
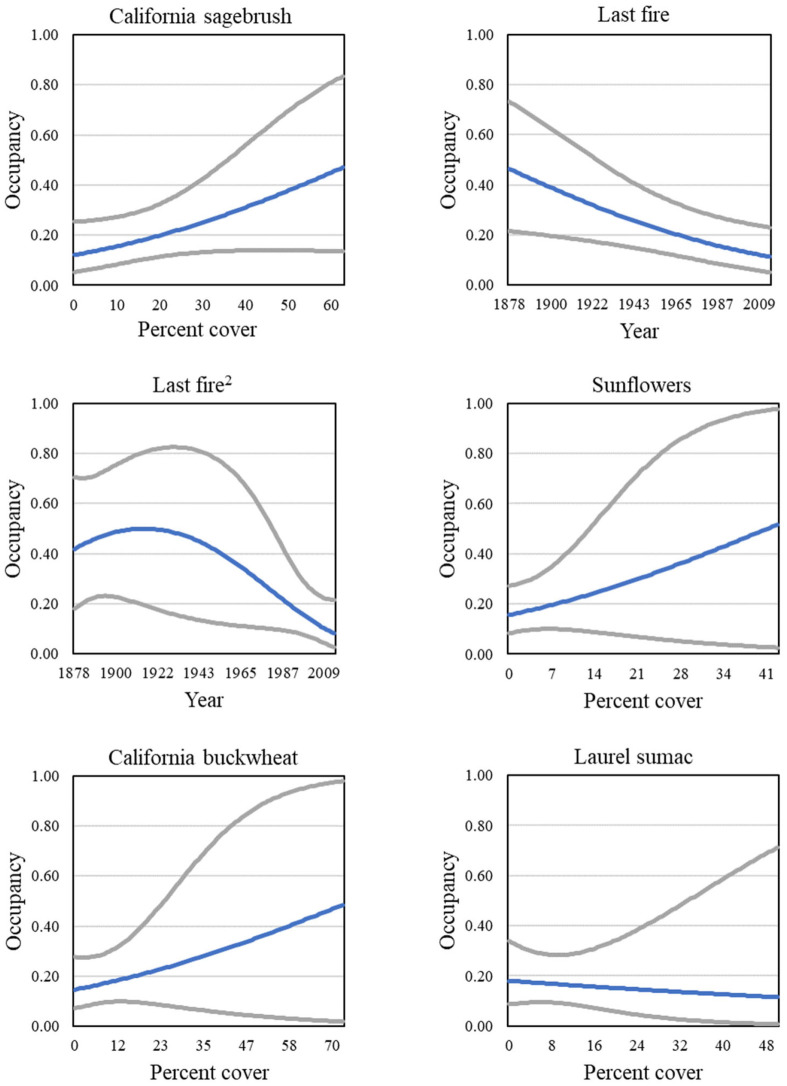
California Gnatcatcher occupancy in relation to cover of California sagebrush, time since last fire (linear and quadratic functions; plotted as year of last fire), sunflowers, California buckwheat, and laurel sumac at San Diego subregional points in 2016. Gray lines represent 95% confidence interval. California sagebrush from model 1, time since last fire from model 2, sunflowers from model 4, California buckwheat from model 5, and laurel sumac from model 7; Table 5).

**Fig 13:**
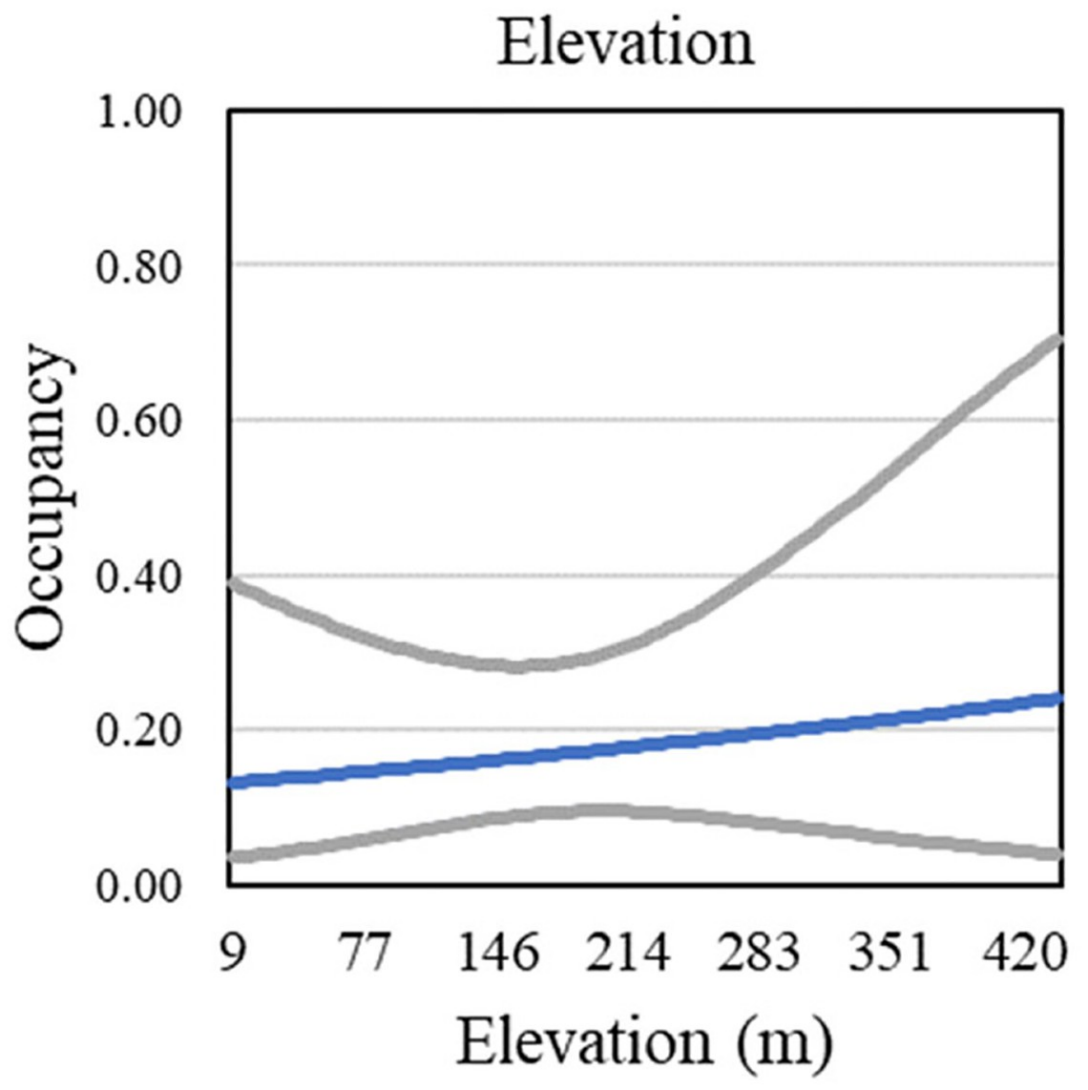
California Gnatcatcher occupancy in relation to elevation at San Diego subregional points in 2016. Gray lines represent 95% confidence interval. From model 6; Table 5).

Three vegetation covariates contributed to well-supported models predicting gnatcatcher occupancy within the San Diego subregion, and a fourth received some support (ΔQAIC_c_ close to 2; Tables 5 and 8). Cover of California sagebrush and time since last fire were the strongest predictors of occupancy, with LASTFIRE appearing in every model as either a linear or quadratic variable (Fig 12). Three covariates appeared in one model each, including California buckwheat and sunflowers, both of which had a positive effect on occupancy, and laurel sumac, which had a negative effect (Fig 12). Occupancy increased slightly with elevation (Fig 13).

**Table 5:** Logistic regression models of California Gnatcatcher occupancy in 2016 as a function of habitat covariates in the San Diego subregion.

	Model	ΔQAIC_c_	AIC_c_ Weight	logLik	Number Parameters	QDeviance
1	ARCA+**LASTFIRE**	0.00	0.21	1.00	4	118.38
2	ARCA+LASTFIRE^2^	0.96	0.13	0.62	5	117.23
3	LASTFIRE^2^	1.25	0.11	0.54	4	119.63
4	ARCA+SUNFL+**LASTFIRE**	1.29	0.11	0.53	5	117.55
5	ARCA+ERFA+**LASTFIRE**	1.47	0.10	0.48	5	117.74
6	ARCA+**LASTFIRE**+ELEV	1.92	0.08	0.38	5	118.18
7	ARCA+MALA+**LASTFIRE**	2.02	0.08	0.36	5	118.29
8	ARCA+**LASTFIRE**+DISTCOAST	2.12	0.07	0.35	5	118.38
9	ARCA+ERFA+SUNFL+**LASTFIRE**	3.27	0.04	0.20	6	117.39
10	ARCA+ERFA+**LASTFIRE**+DISTCOAST	3.49	0.04	0.18	6	117.61
11	ARCA+MALA+TOTSHRTRE+**LASTFIRE**	3.80	0.03	0.15	6	117.92

Detection probability (*p*) constant across models. Variance inflation factor (cĉ) = 2.3. Models are ranked from best to worst based on Akaike’s Information Criterion for small samples (QAIC_c_), the difference between the model’s QAIC_c_ and the highest-ranked model’s QAIC_c_ (ΔQAIC_c_), and AIC_c_ weights. QAIC_c_ is based on -2 x log_e_ likelihood (logLik) and the number of parameters in the model. Minimum QAIC_c_ = 126.61. Only models with ΔQAIC_c_ ≤ 4 presented. Bold means 95% confidence interval of the beta estimate for covariate does not include 0. ARCA = California sagebrush, LASTFIRE = time since last fire, MALA = laurel sumac, SUNFL = sunflowers, ERFA = California buckwheat, DISTCOAST = distance to coast, ELEV = elevation, TOTSHRTRE = total shrub/tree cover. A superscript 2 in a covariate code represents the quadratic (squared) function of that covariate.

#### Orange County subregion

**Fig 14:**
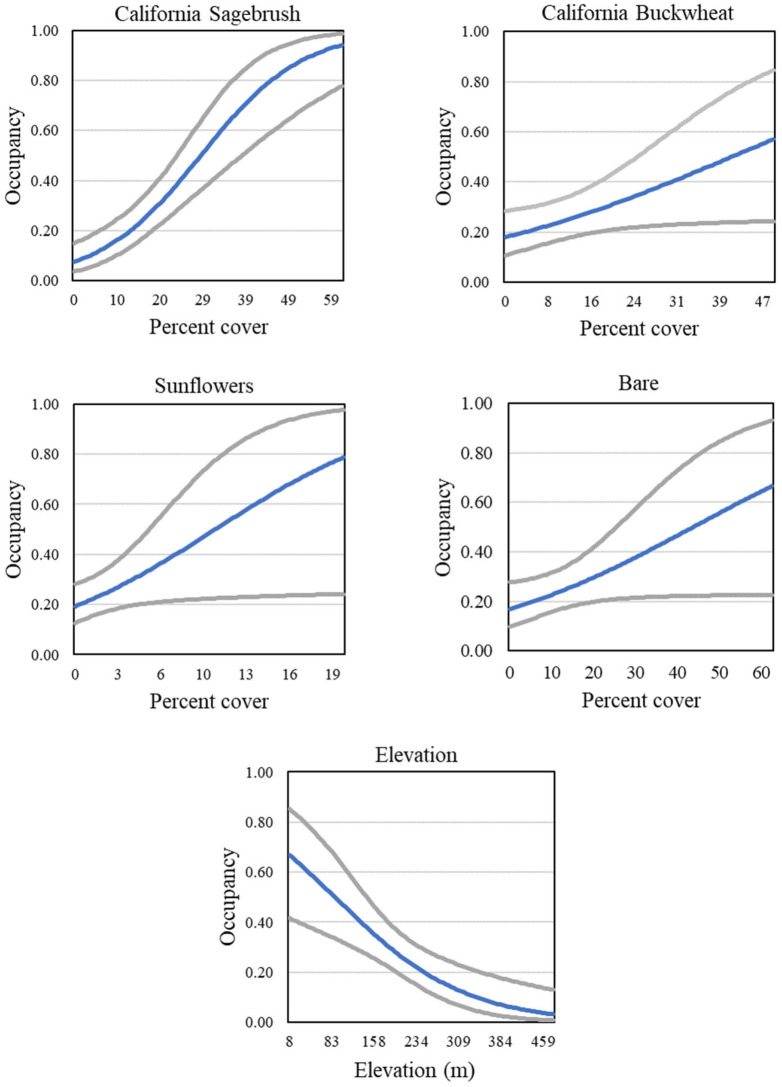
California Gnatcatcher occupancy in relation to cover of California sagebrush, California buckwheat, sunflowers, and bare ground, and to elevation, at Orange County subregional points in 2016. Gray lines represent 95% confidence interval. From model 1; Table 6).

Gnatcatcher occupancy in the Orange County subregion was best described by a model (Table 6) that included cover of California sagebrush, California buckwheat, sunflowers, and bare ground (Fig 14, Table 8), all of which had positive effects on occupancy. Also included in the best supported models was elevation, which unlike in San Diego County was a negative, and stronger, predictor of occupancy (Fig 14).

**Table 6:** Logistic regression models of California Gnatcatcher occupancy in 2016 as a function of habitat covariates in the Orange County subregion.

	Model	ΔAIC_c_	AIC_c_ Weight	logLik	Number Parameters	Deviance
1	**ARCA**+**ERFA**+**SUNFL**+BARE+**ELEV**	0.00	0.44	1.00	7	339.58
2	**ARCA**+ERFA+**SUNFL**+**ELEV**	1.70	0.19	0.43	6	343.45
3	**ARCA**+**ERFA**+**BARE**+**ELEV**	2.19	0.15	0.33	6	343.94
4	**ARCA**+ERFA+**SUNFL**+TOTHRB+**ELEV**	3.08	0.09	0.21	7	342.66
5	**ARCA**+ERFA+**SUNFL**+MALA+TOTHRB+**ELEV**	3.60	0.07	0.17	8	340.99
6	**ARCA**+ERFA+**SUNFL**+**ELEV**+LASTFIRE	3.83	0.06	0.15	7	343.41

Detection probability (*p*) constant across models. Models are ranked from best to worst based on Akaike’s Information Criterion for small samples (AIC_c_), the difference between the model’s AIC_c_ and the highest-ranked model’s AIC_c_ (ΔAIC_c_), and AIC_c_ weights. AIC_c_ is based on -2 x log_e_ likelihood (logLik) and the number of parameters in the model. Minimum AIC_c_ = 354.24. Only models with ΔAIC_c_ ≤ 4 presented. Bold means 95% confidence interval of the beta estimate for covariate does not include 0. ARCA = California sagebrush, ERFA = California buckwheat, SUNFL = sunflowers, BARE = bare ground, ELEV = elevation, TOTHRB = total herbaceous, MALA = laurel sumac, LASTFIRE = time since last fire.

#### Postfire

**Fig 15:**
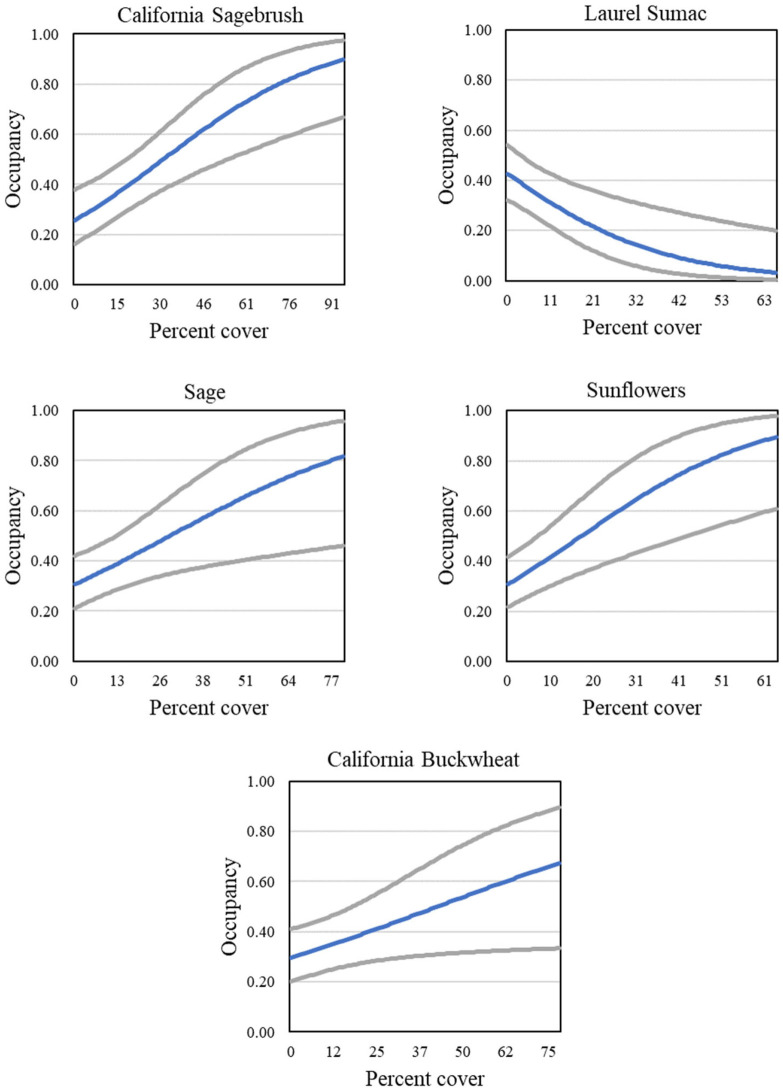
California Gnatcatcher occupancy in relation to cover of California sagebrush, laurel sumac, sage, sunflowers, and California buckwheat at unburned points in 2016. Gray lines represent 95% confidence interval. California sagebrush, laurel sumac, sage, and sunflowers from model 1; California buckwheat from model 2; Table 7).

**Fig 16:**
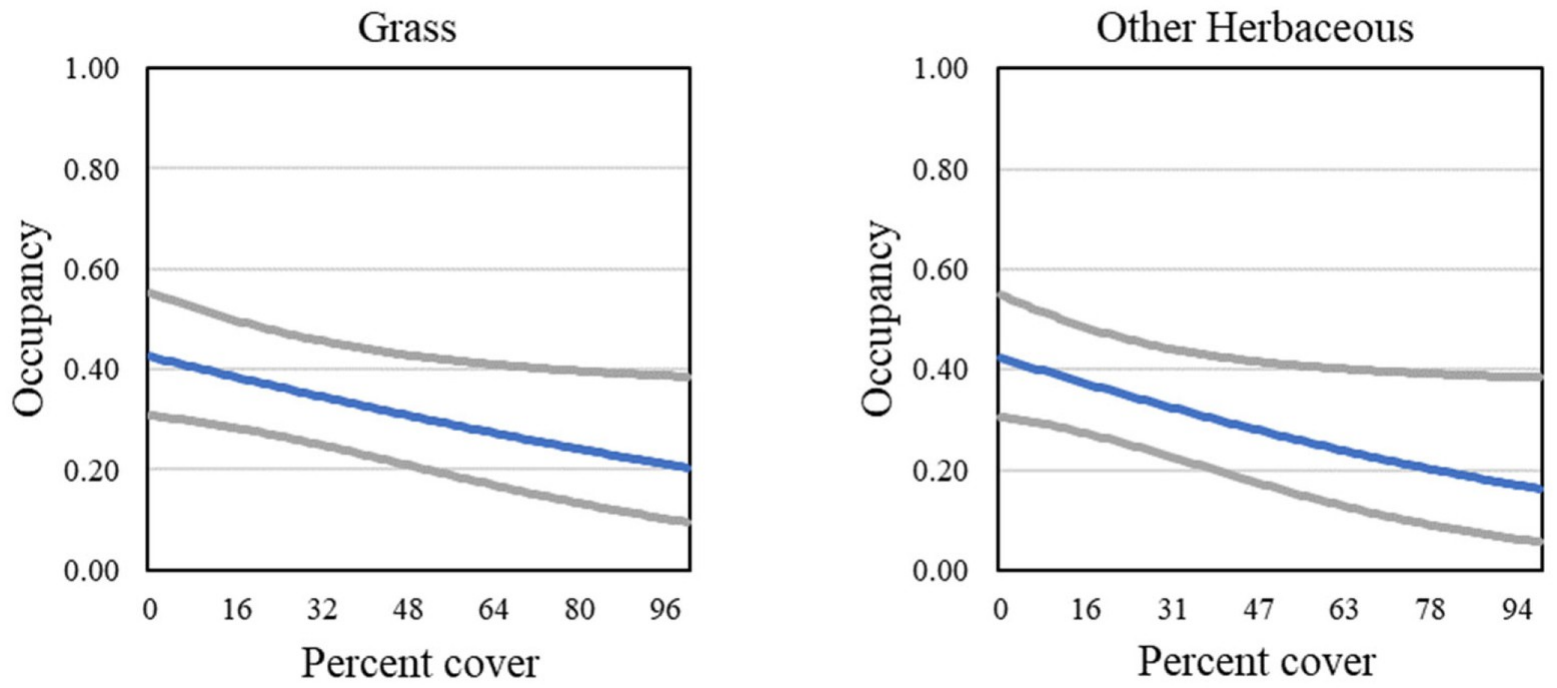
California Gnatcatcher occupancy in relation to cover of grass and other herbaceous at unburned points in 2016. Gray lines represent 95% confidence interval. Grass from model 2; other herbaceous from model 3; Table 7).

**Fig 17:**
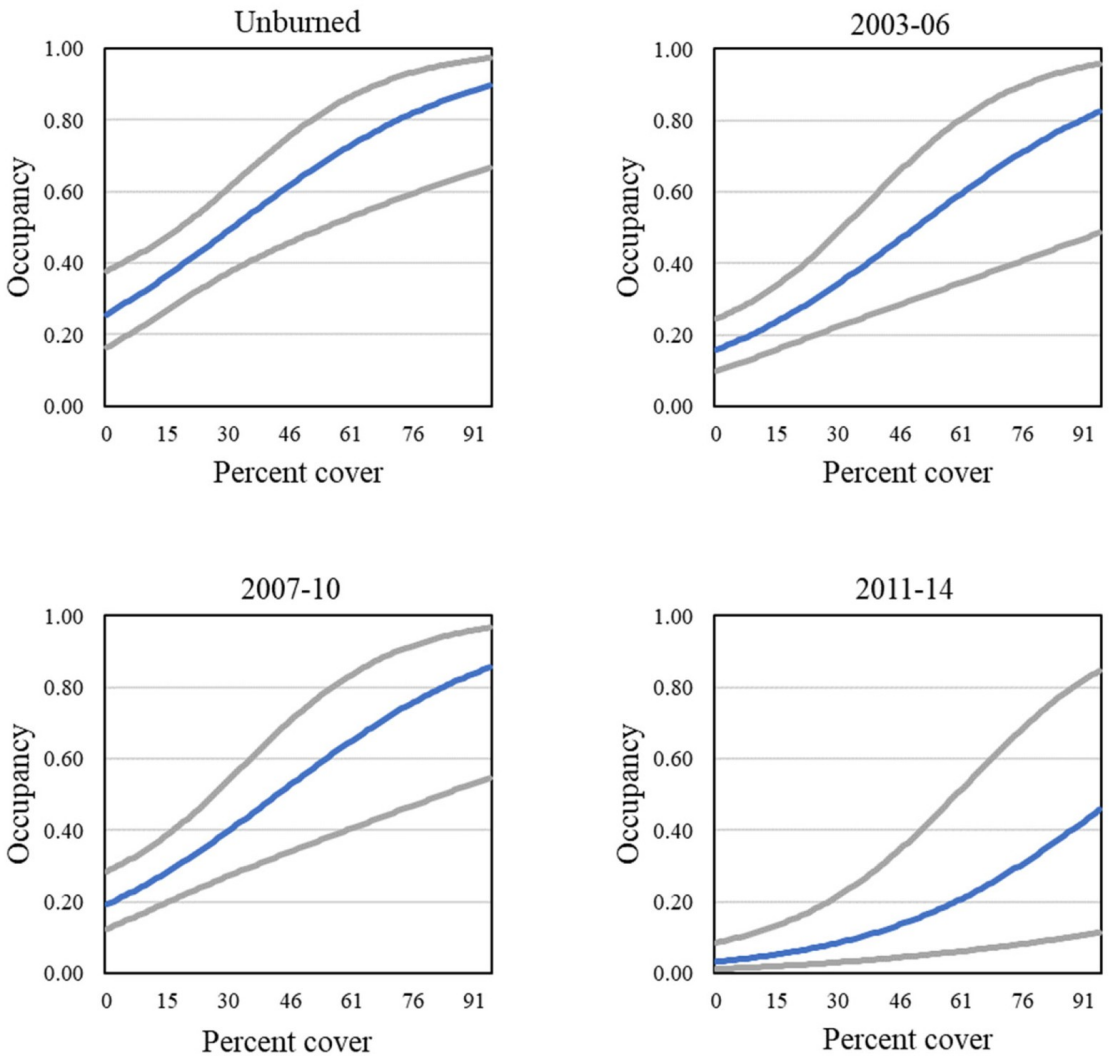
California Gnatcatcher occupancy in relation to cover of California sagebrush in four postfire categories in 2016. Gray lines represent 95% confidence interval. From model 1; Table 7).

Four models describing occupancy in postfire habitat received strong support (Tables 7 and 8). The postfire analysis included four groups (unburned, 2003–06 burned, 2007–10 burned, and 2011–14 burned; for illustrative purposes, results for the unburned group are displayed in figures). The positive effects of California sagebrush and sunflowers, as well as the negative effect of laurel sumac, were in every model (Fig 15). Strong support was found for models with two additional covariates, cover of grass and cover of other herbaceous vegetation (Fig 16), both of which had a negative effect on occupancy. Sage (all black sage in this dataset) cover emerged as a new predictor not seen in analyses at the regional and subregional scales, with a positive effect on occupancy (Fig 15). Relationships between individual covariate predictors and occupancy were similar across groups within the postfire analysis (example shown for California sagebrush, Fig 17), and were stronger than those found for the San Diego subregion as a whole.

**Table 7:** Logistic regression models of California Gnatcatcher occupancy in 2016 as a function of habitat covariates in postfire habitat.

	Model	ΔAICc	AICc Weight	logLik	Number Parameters	Deviance
1	**ARCA**+**MALA**+**SAGE**+**SUNFL**	0.00	0.29	1.00	9	719.40
2	**ARCA**+**MALA**+**ERFA**+**SUNFL**+**GRASS**	0.34	0.25	0.85	10	717.64
3	**ARCA**+**MALA**+**ERFA**+ **SUNFL**+**OTHHRB**	0.57	0.22	0.75	10	717.87
4	**ARCA**+**MALA**+SAGE+**SUNFL**+GRASS+OTHHRB	1.62	0.13	0.45	11	716.81
5	**ARCA**+**MALA**+ERFA+**SUNFL**	2.82	0.07	0.24	9	722.23

Detection probability (*p*) constant across models. Models are ranked from best to worst based on Akaike’s Information Criterion for small samples (AIC_c_), the difference between the model’s AIC_c_ and the highest-ranked model’s AIC_c_ (ΔAIC_c_), and AIC_c_ weights. AIC_c_ is based on -2 x log_e_ likelihood (logLik) and the number of parameters in the model. Minimum AIC_c_ = 737.83. Only models with ΔAIC_c_ ≤ 4 presented. Bold means 95% confidence interval of the beta estimate for covariate does not include 0. ARCA = California sagebrush, MALA = laurel sumac, SAGE = sage (= black sage in this dataset), SUNFL = sunflowers, ERFA = California buckwheat, GRASS = non-native grass, OTHHRB = other herbaceous.

**Table 8:** Comparison of covariates in best supported models (ΔAIC_c_ or ΔQAIC_c_ ≤ 2) predicting California Gnatcatcher occupancy in 2016 in the regional, San Diego (SD) subregional, Orange County (OC) subregional, and postfire datasets. Effect of covariate: “+” = positive, “-”= negative, “+/-”= a threshold effect where the initial effect is positive, “-/+” = a threshold effect where the initial effect is negative. Asterisk means 95% confidence interval of the beta estimate for covariate does not include 0 in at least one model. Parentheses mean that ΔAIC_c_ or ΔQAIC_c_ > 2; see Tables 4 and 5.

Covariate	Regional	SD Subregion	OC Subregion	Postfire
California sagebrush	+*	+	+*	+*
California buckwheat	+*	+	+*	+*
Laurel sumac		(-)		-*
Sunflowers		+	+*	+*
Star thistle				
White sage				
Dead				
Sage				+*
Total herbaceous	-*			
Other herbaceous				-*
Grass				-*
Total shrub/tree	+/-*			
Bare			+	
Shrub height	(+)			
Slope				
Elevation		**+**	-*	
Time since last fire	+/-*	+/-*		

Effect of covariate: “+” = positive, “-”= negative, “+/-”= a threshold effect where the initial effect is positive, “-/+” = a threshold effect where the initial effect is negative. Asterisk means 95% confidence interval of the beta estimate for covariate does not include 0 in at least one model. Parentheses mean that ΔAIC_c_ or ΔQAIC_c_ > 2; see Tables 4 and 5.

#### California Gnatcatcher occupancy

**Fig 18:**
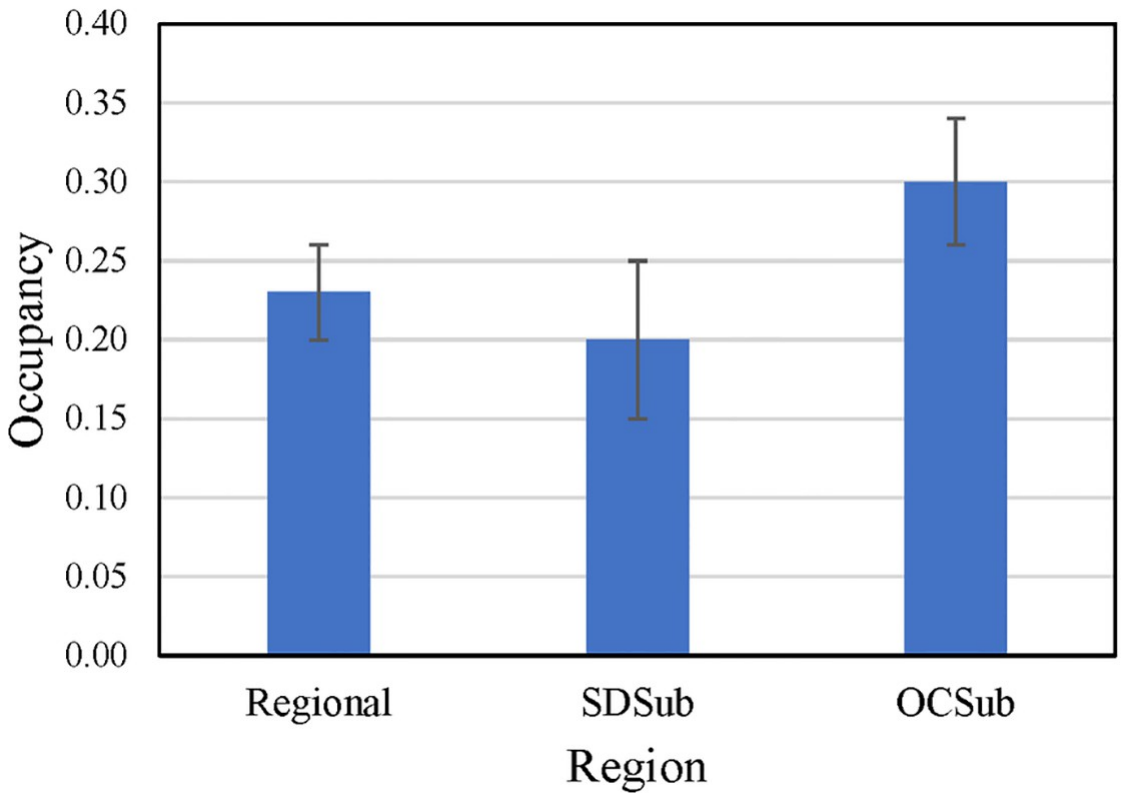
Occupancy (± SE) of California Gnatcatchers at regional and subregional points in 2016. Occupancy estimated as model-averaged occupancy across best supported models (ΔAIC_c_ or ΔQAIC_c_ ≤ 2) for each dataset; see Tables 4–6.

Gnatcatcher occupancy differed across regional and subregional scales in 2016 and was higher in the Orange County subregion than in the San Diego subregion and the region as a whole (Fig 18). Detectability (*p*) ranged from 0.65 at the regional points to 0.69 at the Orange County subregional points.

**Fig 19:**
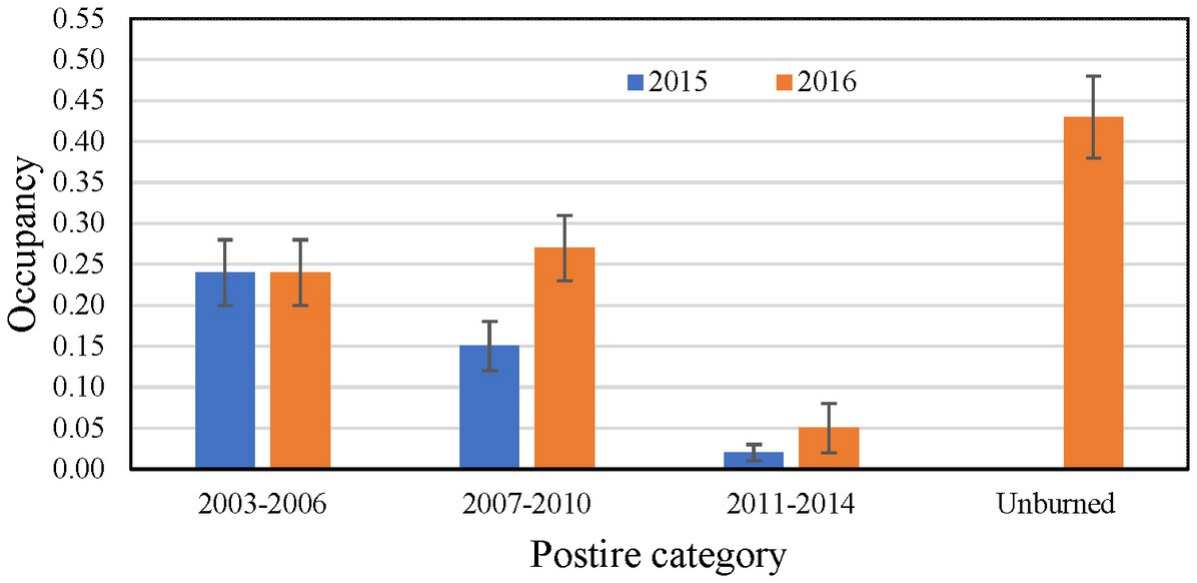
Occupancy (± SE) of California Gnatcatchers as a function of time since burn in 2015 and 2016. Unburned points were not surveyed in 2015. Occupancy in 2015 estimated from model with constant occupancy and detection probabilities. Occupancy in 2016 estimated from model-averaged occupancy across best supported models (ΔAIC_c_ ≤ 2) for postfire dataset; see Table 7.

In burned habitat, Gnatcatcher occupancy varied with time since fire (Fig 19). In 2015, occupancy was highest in habitat burned the longest ago (2003–06), where gnatcatchers occupied 24 percent of points. Occupancy of points burned in 2007–10 was lower, at 15 percent, while just 2 percent of points last burned in 2011–14 were occupied. Detectability was high, ranging from 0.62 at points burned in 2003–06 to 0.74 at points burned in 2007–10.

By 2016, occupancy had doubled in the two most recent burn categories, but was unchanged in the oldest burn category. Occupancy of unburned habitat, at 43 percent, approached twice that in the older burned habitat. Detectability in 2016 was estimated at 0.76, 0.67, and 0.38 for points burned in 2003–06, 2007–10, and 2011–14, respectively, and at 0.73 for unburned points.

## Discussion

This study represents the first effort to characterize habitat condition and gnatcatcher occupancy throughout the entire species’ range in southern California. Our investigation documented variability in structure and composition of coastal sage scrub vegetation that influenced occupancy by gnatcatchers. Our findings provide a rangewide perspective that will help guide gnatcatcher management and recovery at multiple spatial scales.

Rangewide, California sagebrush dominated the woody vegetation at our plots, with California buckwheat, black and purple sage, and laurel sumac making up most of the remaining shrub cover. However, most of the cover at plots was made up of non-native grasses and other herbaceous vegetation, reflecting a history of disturbance associated with fire and possibly other factors such as grazing and pollution [4, 11, 12, 46, 47]. Grass and herbaceous cover at regional plots was over 40 percent higher than at unburned plots, suggesting that recent fires are a primary driver of habitat disturbance in our study area. We found vegetation differences at the subregional scale indicative of differences in recent fire history: in Orange County, where less than 4 thousand ha encompassing 19 percent of suitable (HSI ≥ 0.50) gnatcatcher habitat had burned within 13 years of this study, overall shrub/tree cover was higher, and herbaceous cover lower, than in San Diego, where three large fires burned 35,585 ha of suitable habitat (56%) during the same period. Grass and herbaceous cover in the San Diego subregion was twice as high as in unburned habitat, reflecting the extent of the impact of fire on gnatcatcher habitat in this part of its range.

Fire altered the landscape in ways that were associated with time since burn. Within the first 5 years postfire, sites were dominated by grass and herbaceous vegetation and supported little shrub cover. Once established, grass cover increased over time, and even at points 13 years postfire was still twice as high as that in unburned habitat. Recovery of woody vegetation was underway by 9 years postfire, but it was not until 13 years postfire that total shrub/tree cover was comparable to that in unburned habitat, although species composition differed between burned and unburned sites. Among species, California buckwheat increased rapidly, and by 13 years postfire, cover of buckwheat exceeded that in unburned habitat. California sagebrush cover increased initially, but changed little between 9 and 13 years postfire, at which time it was still only 60 percent of that in unburned habitat. Laurel sumac and black sage, characterized as vigorous resprouters after fire [48], also increased rapidly. Laurel sumac cover exceeded that in unburned habitat within 5 years postfire, and was over twice as high by 9 years postfire. Black sage increased more slowly, but by 13 years postfire, was comparable in cover to unburned habitat. These patterns of rapid postfire increase in non-native annual grasses and slow increase in shrub cover are consistent with findings of previous studies and create the conditions that can lead to vegetation type conversion [3, 4, 11, 12, 49].

Gnatcatcher occupancy varied across the region and was related to fire history. Occupancy was highest in unburned habitat, reflecting the effect of fire at different spatial and temporal scales. At the subregional scale, occupancy was higher in Orange County than in San Diego County, mirroring differences in vegetation composition associated with different fire histories that affect habitat suitability for gnatcatchers. Not only have fires in San Diego County burned more acreage of suitable habitat than in Orange County (see above), they have been more recent, with the median time since last fire at San Diego County points equaling 2007 compared to that of 1993 at Orange County plots. LASTFIRE emerged as the primary predictor of occupancy in the San Diego subregion, reflecting the effects of large and recent fires on vegetation attributes that predicted occupancy in the Orange County and regional datasets, such as woody cover of shrubs favored by gnatcatchers.

At the local scale, occupancy in 2015 was related to time since fire as predicted, with the lowest occupancy in recently burned sites and the highest in habitat burned longest ago (2003–2006). This pattern was observed again in 2016, with occupancy in unburned habitat higher than that in any other postfire category. However, unexpectedly, occupancy in the two intermediate fire categories (2003–06, 2007–10) differed little from one another in 2016. While occupancy in the two most recently burned categories approximately doubled between 2015 and 2016, occupancy in the 2003–06 category was stable across the two years. This could be evidence of a non-linear relationship between occupancy and time since burn, as suggested by our analysis of LASTFIRE at the regional and subregional scales, or other factors limiting occupancy of otherwise suitable habitat. Our habitat assessment revealed little difference in vegetation structure and composition between the 2003–06 and 2007–10 postfire sites, suggesting that something other than habitat condition is limiting occupancy, such as recolonization potential. Winchell and Doherty [19] proposed that recolonization of burned areas proceeds from the perimeter inward, and is dependent on proximity to a source of colonizers. Burned areas close to occupied habitat are likely to be recolonized faster than those more distant from a source of colonizers [50]. Differences between our two postfire categories in this regard warrant further investigation.

Previous studies have shown that gnatcatchers can recolonize burned sites within 2 to 5 years near the coast [50, 51], and 5–10+ years inland, depending on fire intensity [52] and conditions affecting vegetation recovery like precipitation, temperature, and aspect [53]. We observed gnatcatchers occupying habitat near the coast that had burned two years previously. While it is possible that recolonization can begin quickly as patches of suitable habitat emerge, occupancy will be limited by available suitable habitat. We found that it took at least 9 years postfire for woody vegetation to attain the structure and cover favored by gnatcatchers, and our modelled relationships between occupancy and LASTFIRE at the regional and subregional scales suggest it may take decades for burned coastal sage scrub to achieve occupancy levels typical of unburned habitat. However, we caution that this conclusion is based on analysis of a variable (LASTFIRE) obtained from a spatial database of fire perimeters (CalFire) that may be incomplete, and thus this conclusion should be considered preliminary. Several investigators have used CalFire data to examine the effects of wildfire on vegetation in southern California [4, 6, 54], acknowledging the shortcomings of the database. In particular, small fires (< 25 ha) may be missed, and perimeters may not be accurate. These errors grow the further back in time the records go, and many studies use 1919 as the cutoff for reliable data [55–59] or use a more recent subset of the data [60]. We used records dating back to 1878 in order to assign a value for LASTFIRE to every point in our regional and subregional datasets and thereby meet our samples size requirements for detecting differences in occupancy. In our application, missed fires, especially before 1919, would cause us to over-estimate the length of time since a particular point burned, and thus over-estimate the modelled time required to achieve occupancy comparable to that in unburned habitat. Although our regional and Orange County subregional datasets may be subject to this potential bias, we consider our analysis of LASTFIRE for San Diego County to be robust as only 1 value for LASTFIRE in that dataset preceded 1919, and only 8 preceded 1950. We have high confidence in our postfire dataset where we analyzed recovery of points that burned since 2003, and compared them to an “unburned” category of points that last burned before 2002 where the potential bias created by missed fires would not affect our results. We suggest that continued monitoring of the postfire points will yield valuable insights into the recovery process during the first few years and decades following fire.

Gnatcatchers occupied habitat non-randomly, and modelling occupancy using habitat covariates clarified bird-habitat relationships. Cover of California sagebrush was the strongest predictor of gnatcatcher occupancy, and appeared in every dataset. Previous studies have documented the association of gnatcatchers with sagebrush-dominated sage scrub [7, 61], and recent modelling of gnatcatcher occupancy in unburned habitat in San Diego County [20] identified California sagebrush as a positive predictor. In our study, gnatcatcher occupancy at regional and subregional scales increased with increasing cover of California sagebrush up to about 40–60 percent, similar to the relationship documented by Winchell and Doherty [20] for occupied habitat.

California buckwheat was another strong predictor of gnatcatcher occupancy in all of our datasets. Typically co-occurring with California sagebrush in stands of coastal sage scrub, particularly inland stands [62], California buckwheat has not previously been identified as a predictor of occupancy, although gnatcatcher association with buckwheat co-dominated habitats has been reported [7, 63–65]. We found that gnatcatcher occupancy increased with cover of California buckwheat up to about 50–60 percent at regional and Orange County subregional points. Buckwheat had less influence on occupancy within the San Diego subregion as a whole, but was a very strong predictor of occupancy in postfire habitat, where cover of buckwheat sometimes reached 75 percent. This may explain why California buckwheat did not emerge as a predictor of occupancy in a previous investigation [20] since only unburned sites were included in that analysis.

Sunflowers were a positive predictor of occupancy in the Orange County subregion and postfire sites, with some support in the San Diego subregion. Weaver [65] found high gnatcatcher abundance and density in coastal sites dominated by bush sunflower (*Encelia californica*) and California sagebrush. The inland counterpart, brittlebush (*E*. *farinosa*) co-occurs with California sagebrush and California buckwheat, and is a rapid colonizer of disturbed sites [62]. Gnatcatcher occupancy in the Orange County subregion increased with sunflower cover up to about 20 percent, the highest cover we recorded for this region. At postfire sites, sunflower cover sometimes reached 60 percent, the upper limit on its representation in coastal sage scrub plant communities [62].

Laurel sumac was found to be a significant negative predictor of gnatcatcher occupancy in the postfire dataset, and received some support for an influence in the San Diego subregion. Previously hypothesized to be a positive predictor of occupancy at the landscape scale because its thermal tolerance coincides with that of the gnatcatcher [66], laurel sumac was found instead to be a negative predictor in San Diego County [20]. Laurel sumac resprouts readily after fire, and at our plots co-dominated the recovering woody vegetation along with California sagebrush and California buckwheat. However, relative cover of laurel sumac in postfire sites, at 30 percent of combined cover of sumac, sagebrush and buckwheat, exceeded that in unburned habitat, where sumac makes up less than 20 percent of the combined cover. While providing shrub cover initially, rapid spread of laurel sumac may inhibit the recovery of species more favored by gnatcatchers, and reduce habitat suitability long-term. Our results showed declining occupancy as cover of laurel sumac increased to about 50 percent, above which the probability of occupancy was minimal.

Black sage-dominated habitat in coastal areas has been reported as supporting low abundance of gnatcatchers, although use of such habitats at inland sites may be more extensive [61, 65]. Winchell and Doherty [20] tested for a negative effect of black sage on gnatcatcher occupancy, hypothesizing that the closed canopy of this plant community might limit foraging habitat, but found no relationship. SAGE did not emerge as a predictor in our study at regional or subregional scales; however, it was a strong positive predictor of gnatcatcher occupancy in postfire sites. Like laurel sumac, black sage resprouts quickly after fire, providing a shrub component to the recovering vegetation. However, unlike laurel sumac, at our plots sage cover remained comparable to that in unburned habitat even 13 years postfire, and did not appear to be excluding other shrubs favored by gnatcatchers.

Although we found relationships between gnatcatcher occupancy and cover of individual plant species, coastal sage scrub typically includes a mix of shrub and tree species, each contributing to overall habitat suitability. Regionally, occupancy was predicted by total shrub/tree cover in a non-linear relationship. Occupancy increased with total shrub/tree cover up to about 40 percent, but declined with cover above that. Winchell and Doherty [20] proposed 40 percent cover of California sagebrush as a target for habitat restoration, which in combination with other species would exceed the threshold suggested by our results. Because the two studies differed in geographic location and extent, timeframe, whether burned habitats were sampled (this study) or not [20], and modelling approach, it is not possible to determine whether our apparently different findings are the result of methodological or biological differences. Establishing targets for habitat restoration is an important application of the results of studies describing gnatcatcher-habitat associations, and a topic for further refinement.

Herbaceous vegetation, which dominated all our study plots, negatively influenced gnatcatcher occupancy regionwide. The primary component of herbaceous cover was non-native grass, made more explicit in analysis of the postfire dataset. Postfire invasion of grass may inhibit seedling establishment of shrubs preferred by gnatcatchers [67], changing the structure and composition of coastal sage scrub and in extreme cases leading to type conversion to grasslands [3, 4, 11, 68]. At our postfire plots, grass and other herbaceous vegetation not only established quickly after fires, but increased with time, making it unlikely that it will subside naturally.

We found little support for effects of physical variables on occupancy outside of a negative effect of elevation in the Orange County subregion. There, occupancy declined with increasing elevation over a range of 8–450 m, consistent with findings by Atwood and Bolsinger [69], who found that all of 68 gnatcatcher occurrences in coastal Orange County between 1960 and 1990 were below 500 m in elevation. Winchell and Doherty [13] found a decline in occupancy over a range of 0–1000 m in San Diego County; in contrast, our results produced limited support for a slight positive effect over a narrower range of elevation in the San Diego subregion. Coastal sage scrub vegetation composition varies along gradients of distance to coast, elevation, and slope [48, 62, 70], and it is likely that in our analyses the effects of vegetation covariates outweighed what might be considered indirect effects of these physical variables on gnatcatcher occupancy.

Comparable data for evaluating trends in gnatcatcher occupancy in our regional study area are not available. The most recent information on occupancy was reported by Winchell and Doherty [13], who estimated occupancy for unburned habitat in San Diego County in 2009 at approximately 41–44 percent for high and very high quality habitat, respectively. Habitat in these quality classes best corresponds to our unburned category, for which we estimated occupancy in 2016 at 43 percent. Future surveys using our standardized geographic scope and protocol will facilitate a more rigorous evaluation of population trends.

Collectively, our rangewide results reveal a widespread and long-term impact of wildfire on California Gnatcatcher habitat, particularly in San Diego County. We documented habitat associations that allow a determination of specific ways that fire affects habitat suitability. These data provide a baseline from which future monitoring can evaluate changes in habitat condition over time, and improve our understanding of the factors influencing gnatcatcher occupancy. This in turn will guide development of management practices, particularly pre- and postfire management, to ensure the long-term persistence of California Gnatcatchers in southern California.
